# Intrahost HA polymorphisms and culture adaptation shape antigenic profiles of H3N2 influenza viruses

**DOI:** 10.1128/jvi.01775-25

**Published:** 2026-01-07

**Authors:** Kritika Prasai, Zunlin Yang, Minhui Guan, Tao Li, Daphne Ware, Jun Hang, Xiu-Feng Wan

**Affiliations:** 1 NextGen Center for Influenza and Emerging Infectious Diseases, University of Missouri 14716, Columbia, Missouri, USA; 2 Department of Electrical Engineering & Computer Science, College of Engineering, University of Missouri 199679, Columbia, Missouri, USA; 3 Bond Life Sciences Center, University of Missouri 14716, Columbia, Missouri, USA; 4 Department of Molecular Microbiology and Immunology, University of Missouri 219013, Columbia, Missouri, USA; 5 Viral Diseases Branch, Walter Reed Army Institute of Research 8394, Silver Spring, Maryland, USA; 6 Mississippi Public Health Laboratory, Jackson, Mississippi, USA; Emory University School of Medicine, Atlanta, Georgia, USA

**Keywords:** influenza A virus (H3N2), vaccine strain selection, antigenic characterization, intrahost polymorphism, adaptive mutation, purifying selection, MDCK cells, serological assays

## Abstract

Accurate antigenic characterization of influenza viruses is critical for vaccine strain selection but is often confounded by intrahost genetic diversity and culture-induced adaptations. We analyzed 60 A(H3N2)-positive nasopharyngeal specimens collected during the 2017–2018 influenza season to determine how virus propagation in MDCK cells affects viral genetic and antigenic properties. Deep sequencing revealed frequent genome-wide intrahost polymorphisms, including amino acid variants within major hemagglutinin (HA) antibody-binding sites. Virus propagation imposed rapid purifying selection, markedly reducing intrahost genetic diversity. Serological analyses demonstrated that these selective events altered antigenic properties, indicating that culture adaptation can alter antigenic profiles. To assess the functional impact of HA polymorphisms, we generated mixed viral populations containing defined ratios of HA-160K, HA-160T, and HA-160I variants identified in clinical samples. These reconstituted populations exhibited a continuum of antigenic reactivities, confirming that amino acid polymorphisms drive antigenic heterogeneity among clinical samples. Together, our findings demonstrate that intrahost variation contributes to antigenic diversity in human A(H3N2) clinical specimens and that virus isolation procedures can bias antigenic characterization, underscoring the importance of direct profiling of uncultured clinical material to improve vaccine strain selection accuracy for seasonal human influenza.

**IMPORTANCE:**

Accurate antigenic characterization of influenza viruses is essential for vaccine strain selection, yet routine isolation of viruses in cell culture can introduce genetic changes that obscure the properties of circulating strains. By combining deep sequencing with serological analysis of clinical specimens and cultured isolates, we demonstrate that virus propagation of human seasonal A(H3N2) in MDCK cells imposes strong purifying selection and alters antigenic profiles. Furthermore, we show that minor amino acid polymorphisms present in clinical samples can generate measurable antigenic diversity, emphasizing that natural intrahost variation shapes antigenic outcomes. These findings reveal a critical source of bias in antigenic characterization workflows and underscore the importance of directly assessing uncultured clinical material. Improved understanding of how culture adaptation and intrahost genetic diversity influence antigenic data will advance knowledge of antigenic evolution in circulating influenza viruses and improve the accuracy of vaccine strain selection for human seasonal influenza.

## INTRODUCTION

Seasonal influenza A viruses (IAVs) remain a continuing global public health challenge, causing frequent outbreaks and disproportionately affecting older adults, young children, and individuals with underlying health conditions (1–3). Although influenza vaccines are updated annually to match circulating strains, vaccine effectiveness (VE) has been consistently suboptimal, with pooled estimates in the Northern Hemisphere averaging only ~22%, and typically below 30% for outpatient settings in recent seasons (4–6). One major contributing factor is antigenic mismatch between vaccine strains and circulating viruses, driven both by rapid viral evolution and limitations in vaccine strain selection and virus propagation methodologies (7–10).

Global influenza control depends on the biannual vaccine strain selection process coordinated by the World Health Organization (WHO) Global Influenza Surveillance and Response System (GISRS), which integrates global virological surveillance and antigenic characterization (8, 11). However, most antigenic data are derived from viruses isolated and amplified in laboratory systems, such as embryonated chicken eggs or immortalized cell lines (e.g., MDCK and MDCK-derived variants) (12, 13). Routine assays, including hemagglutination inhibition (HAI) and microneutralization (MN), generally require high-titer cultured isolates and are rarely performed directly on uncultured clinical material. As a result, antigenic measurements that guide vaccine strain selection can be influenced not only by the antigenic properties of viruses circulating in humans but also by selection pressures imposed during virus isolation and passaging (14). These systems impose selection pressures that can introduce adaptive mutations, especially in the hemagglutinin (HA), altering antigenic epitopes and potentially misrepresenting viruses circulating in humans (15–26). While egg adaptations are well documented (21, 27, 28), the extent to which cell-derived adaptations affect antibody binding sites and antigenic properties remains incompletely understood (29, 30). Deep sequencing has revealed that individual infections often harbor low-frequency intrahost genetic variants (31, 32), yet culture-based virus isolation imposes selection that reshapes variant frequencies, enriching variants, whether initially dominant or minor, that confer a replication advantage in the isolation system, potentially obscuring the *in vivo* antigenic landscape of clinical samples.

In this study, we examined how intrahost genetic polymorphisms shape the antigenic properties of clinical samples and how virus propagation in MDCK-derived cells alters these patterns. Using 60 A(H3N2) confirmed samples collected during the 2017–2018 influenza season, we compared the antigenic properties of viruses in clinical specimens with their corresponding MDCK-derived isolates using polyPLA, a highly sensitive assay capable of directly assessing viral antigenicity from clinical material (33, 34). Unlike conventional serological assays such as HAI or MN assays, which require high-titer cultured isolates and cannot be performed on low-titer samples, polyPLA enables antigenic assessment from low-virus loads (~1,000 TCID₅₀), providing superior sensitivity. We further evaluated how intrahost variation contributes to antigenic heterogeneity and the extent to which culture-based isolation alters this diversity. Together, our findings underscore the importance of characterizing viral populations directly from clinical specimens to better capture antigenic complexity and improve vaccine strain selection.

## RESULTS

### Clinical samples and virus isolation

The 2017–2018 Northern Hemisphere influenza season was dominated by A(H3N2) viruses, with the WHO-recommended H3N2 vaccine component derived from A/Hong Kong/4801/2014 (clade 3C.2a) (4). Nasopharyngeal swab (NPS) specimens were obtained through routine influenza surveillance conducted by the Mississippi Public Health Laboratory from patients presenting with influenza-like illness across a statewide network. Samples were selected based on confirmed A(H3N2) qRT-PCR positivity and sufficient residual volume for both virus isolation and whole-genome sequencing and were not further clinically stratified.

To access how intrahost genetic variation and cell culture adaptation influence the genetic and antigenic properties of A(H3N2) influenza viruses, we collected 60 de-identified NPS samples from patients with PCR-confirmed influenza A(H3N2) infection during the 2017–2018 season. Virus isolation was attempted in MDCK-SIAT1 (kindly provided by Dr. Mikhail Matrosovich) (35) and humanized MDCK (hCK cells; kindly provided by Dr. Yoshi Kawaoka). To compare isolation efficiency, 40 clinical specimens were inoculated into both cell lines. After two passages, hCK cells yielded isolates from 36/40 samples (90%), whereas MDCK-SIAT1 recovered only 15/40 (37.5%). Notably, all isolates recovered in MDCK-SIAT1 were also recovered in hCK cells, with no MDCK-SIAT1–unique recoveries. Based on the higher recovery rate in hCK cells, all subsequent genomic and antigenic analyses focused on hCK-derived isolates. Overall, 60 clinical specimens, 45 produced successful hCK isolates, resulting in 45 matched clinical–isolate pairs for downstream analyses.

### Co-circulation of genetically distinct H3N2 lineages during the 2017–2018 influenza season

Viral RNA was extracted from all clinical specimens and isolates and subjected to next-generation sequencing (NGS), yielding 102 genomes after excluding three clinical samples with insufficient coverage. This data set included 57 high-quality clinical genomes and 45 paired isolates.

**Fig 1:**
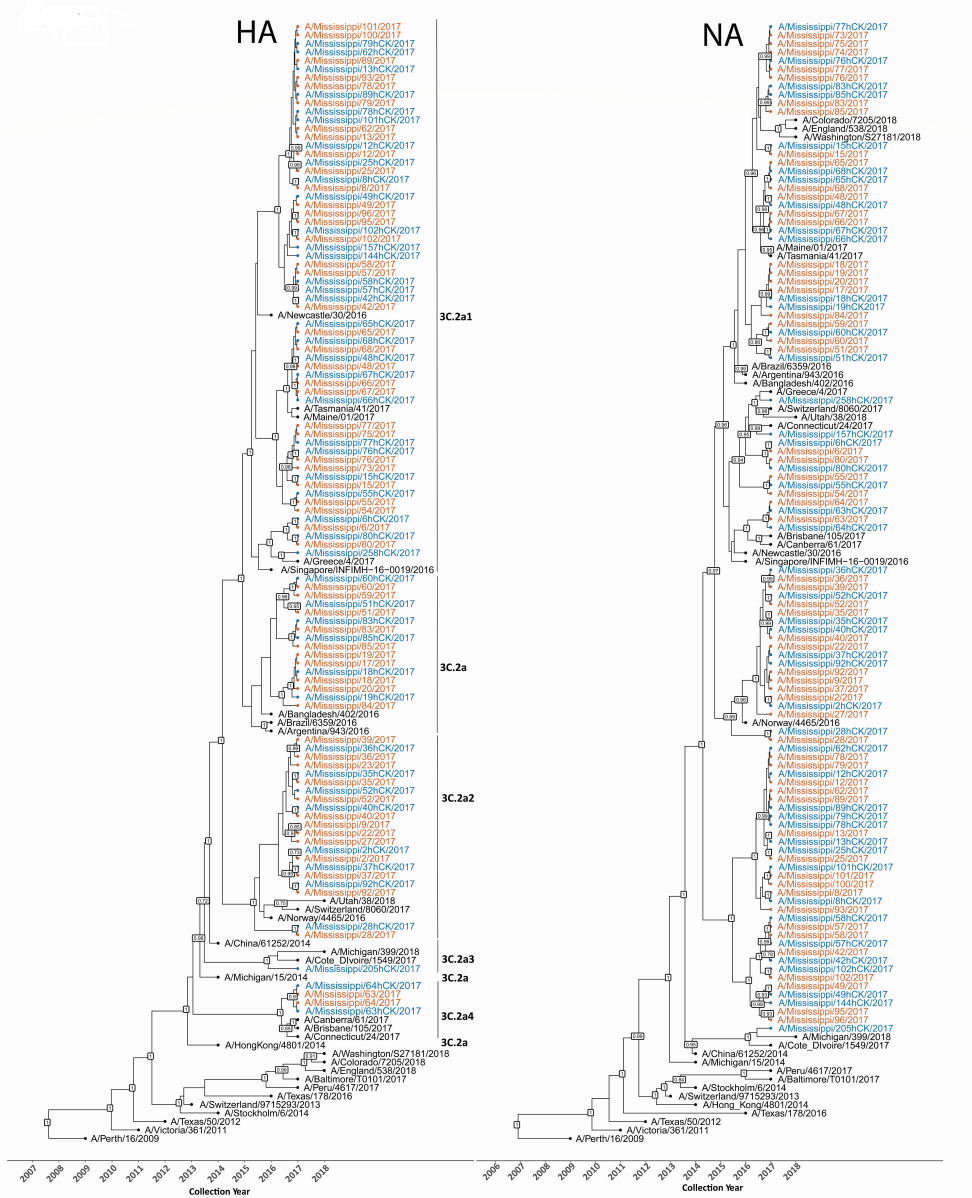
Time-scaled phylogenetic trees of HA and neuraminidase (NA) gene segments of A(H3N2) viruses from the 2017 to 2018 influenza season. The paired clinical samples and isolates are denoted as *clinical sample identifier*-NPS and *clinical sample identifier*-hCK, respectively; Maximum clade credibility (MCC) trees were reconstructed using BEAST v1.10.5.0 (36), where the clinical specimens are shown in red text, their corresponding hCK-derived isolates are shown in blue text, and representative circulating H3N2 strains obtained from the GISAID EpiFlu database (collected up to 2018) are shown in black text. Major clades (3C.2a, 3C.2a1, 3C.2a2, 3C.2a3, and 3C.2a4) are annotated on the right for reference. Posterior probability support values ≥0.7 are displayed at internal nodes. The x-axis denotes collection year, with reference strains (e.g., A/Hong Kong/4801/2014(H3N2) and A/Perth/16/2009 (H3N2)) included for temporal calibration.

HA gene analysis of the 57 clinical genomes revealed clustering into five clades: 3C.2a (*n* = 10, 17.5%), 3C.2a1 (*n* = 32, 56.1%), 3C.2a2 (*n* = 13, 22.8%), 3C.2a3 (*n* = 1, 1.7%), and 3C.2a4 (*n* = 2, 3.5%) with 3C.2a1 being the most prevalent (Fig. 1a). Notably, most circulating viruses were genetically distinct from the vaccine strain A/Hong Kong/4801/2014 (H3N2) (HK/14), which belonged to clade 3C.2a. All virus isolates clustered closely with their paired clinical samples, indicating that the overall genetic composition was largely preserved during virus isolation.

**Fig 2:**
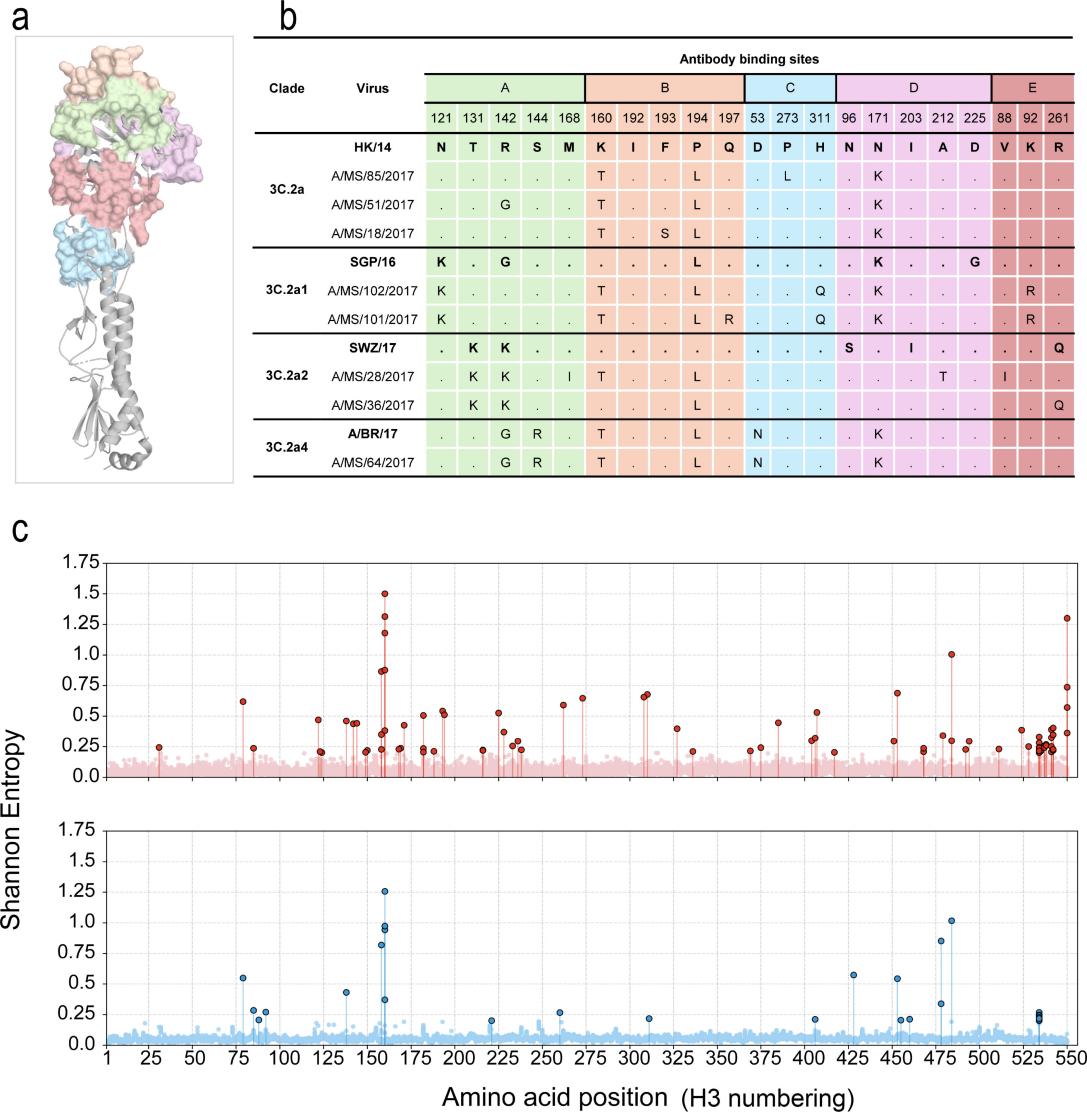
Genetic diversity in the HA genes among A(H3N2) viruses from the 2017 to 2018 influenza season. (**a**) HA trimer structure (PDB: 4WE8) rendered in PyMOL, with the five canonical antigenic sites (A–E) highlighted in distinct colors: Site A (green), Site B (orange), Site C (blue), Site D (purple), and Site E (pink). The structural context illustrates the spatial distribution of residues analyzed for antigenic variation. (**b**) Consensus amino acid diversity across major clades of A(H3N2) viruses. Amino acid differences at antigenic sites (rows = clades, columns = antigenic residues) are shown for the 2017–2018 clinical data set relative to the vaccine strain HK/14, which belongs to clade 3C.2a. Each colored block maps with the structure corresponding to each antigenic site, and substitutions characteristic of clades 3C.2a, 3C.2a1, 3C.2a2, and 3C.2a3 are highlighted. (**c**) Shannon entropy analysis of intrahost variability across the HA gene. Entropy values were calculated from deep sequencing of 41 matched clinical (top panel, red scatter points) and hCK-isolate (bottom panel, blue scatter points) samples. Each point represents the entropy at a single amino acid position. Positions with entropy >0.2 are highlighted as high-variability sites. Entropy values were calculated from iSNVs identified using DiversiTools (https://github.com/josephhughes/DiversiTools).

Within each HA subclade, viruses were genetically similar, but divergence relative to HK/14 was disproportionately concentrated within antibody-binding sites (Fig. 2b). Specifically, clade 3C.2a viruses differed at six positions (i.e., 142, 160, 171, 193, 194, and 273), clade 3C.2a1 at seven positions (i.e., 92, 121, 160, 171, 194, 197, and 311), clade 3C.2a4 at six positions (i.e., 53, 142, 144, 160, 171, and 194), and clade 3C.2a2 showed the highest divergence, with substitutions at positions 88, 92, 131, 142, 160, 168, 194, 212, and 261 (Fig. 2b).

Phylogenetic analyses also showed the viruses circulating during this season exhibited genetic divergence from the vaccine strain used in this season (Fig. 1; Fig. S1).

Overall, these findings demonstrate that multiple H3N2 lineages co-circulated during the 2017–2018 influenza season. Although HK/14, a clade 3C.2a virus, was the recommended vaccine strain, most circulating viruses in this cohort belonged to clades 3C.2a1 and 3C.2a2.

### Cell culture induces adaptive mutations and purifying selection in MDCK-propagated viruses

To assess the impact of virus isolation on genetic diversity, we analyzed NGS data from clinical specimens and their paired isolates to examine intrahost polymorphisms. Entropy analysis revealed substantial within-host diversity, particularly in the HA and NA genes, whereas internal genes, especially the polymerase complex, showed limited variability (Fig. 2c; Table S1; Fig. S2). In the HA gene, diversity was higher and broader in clinical samples than in isolates, especially at five major antibody-binding sites, receptor-binding sites within HA1, and the HA2 stem domain. In contrast, isolates exhibited comparatively fewer polymorphisms, with some high-entropy positions extending into the transmembrane regions.

**Fig 3:**
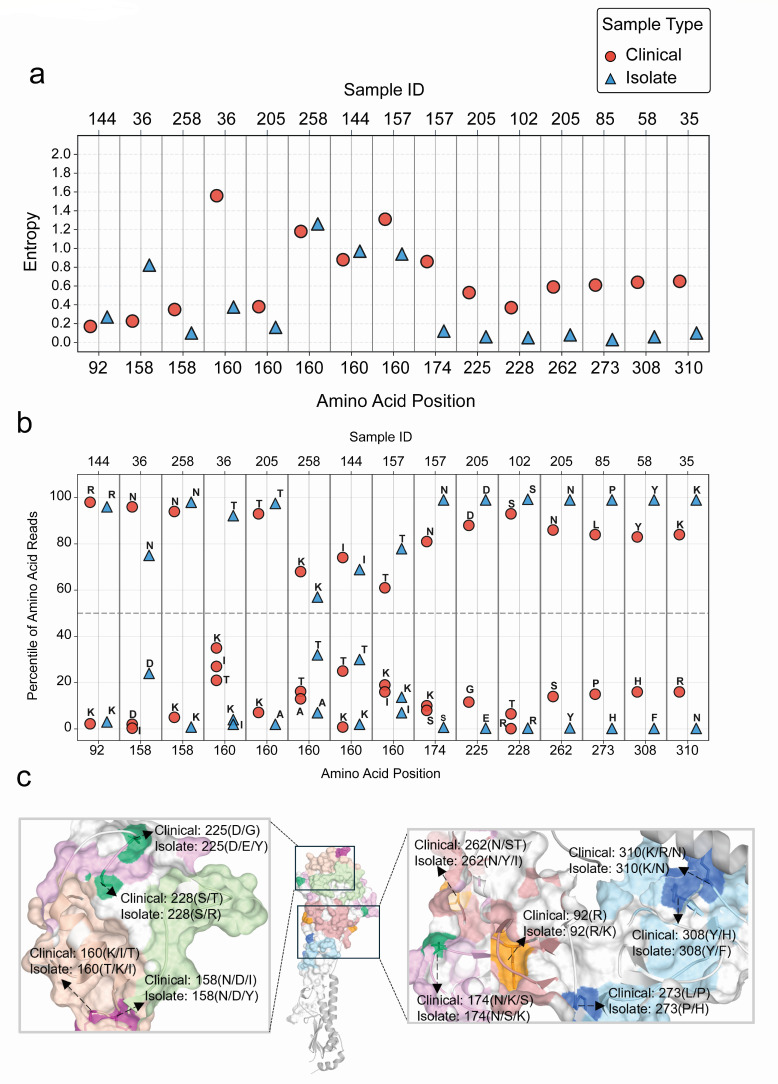
Intrahost polymorphisms at key HA antigenic residues were reduced following culture. (**a**) Shannon entropy values at 10 representative HA residues from paired clinical (red circles) and hCK-derived isolate (blue triangles) samples. (**b**) Amino acid frequency distributions are shown at the same sites for the same samples. Positions with high entropy in panel A (e.g., 36-NPS at residue 160, clinical mixture of K/I/T) correspond to broad distributions of amino acids in clinical samples, whereas low-entropy positions (e.g., 160T in the isolate) correspond to near-fixation of a single residue. Thus, panel B provides the underlying amino acid composition that explains the entropy patterns in panel A. (**c**) Structural mapping of polymorphic residues on the HA trimer (PDB: 4WE8). Variable positions (228, 160, 310, 273, and 308) are highlighted within antigenic sites B, C, and D. Clinical samples exhibited mixtures of residues (e.g., 228 S/T/R; 273 L/P; 308 Y/H; 310 K/R/N), which converged to one or two dominant states in isolates (e.g., 228 S/R; 273 P/H; 308 Y/F; 310 K/N).

Most polymorphic HA residues were surface-exposed and located within or near canonical antibody binding sites, including 158, 160 (antibody binding site B), 273, 308, and 310 (Site C), 172, 225, 228 (site D), 92 and 262 (site E) with additional residues at 385, 484, and 527 (Fig. 3a through c). Across these key positions, clinical samples consistently showed higher site-specific entropy than isolates, indicating multiple co-circulating variants *in vivo*, whereas isolates converged toward one or two dominant residues under purifying selection. For example, at site 160 in clinical sample 36-NPS, three variants (K 35%, I 27%, and T 21%) rapidly approached fixation after passage (T92%; entropy 1.56→0.38). In contrast, in the clinical sample 18-NPS, residue 385 changed from F (91%) in the clinical sample to I (98.8%) after isolation (entropy 0.41→0.04), whereas at residue 484 in clinical sample 25-NPS, an E/G mixture (55/44%) in the clinical samples changed to G (51%) in the isolate but entropy remained high (0.99→1.00), supporting that selection can both fix specific residues and maintain local diversity.

Together, these findings demonstrate that clinical samples exist as heterogeneous viral quasispecies, with genetic variability concentrated at surface-exposed residues, particularly within antibody-binding sites. Virus isolation in cell culture imposes strong purifying selection, favoring adaptive substitutions while reducing or excluding variants representative of *in vivo* viral diversity. Such culture-driven pressures may bias the true antigenic landscape and complicate vaccine strain selection.

### Culture adaptation in MDCK cells alters antigenic profiles of H3N2 influenza viruses

To assess whether changes in intrahost polymorphisms during virus isolation in MDCK cells alter antigenic properties, we analyzed 24 viral isolates with a minimum HA titer of 1:8 using conventional HAI assays (Table 1). In parallel, polyPLA was performed on 16 clinical specimen–isolate pairs representing five genetic clades: 3C.2a (*n* = 5), 3C.2a1 (*n* = 4), 3C.2a2 (*n* = 4), 3C.2a3 (*n* = 1), and 3C.2a4 (*n* = 2) (Fig. 1). Four antigenically distinct reference strains, A/Hong Kong/4801/2014 (HK/14, clade 3C.2a), A/Singapore/IFNIMH-16-0019/2016 (SGP/16, clade 3C.2a1), A/Kansas/14/2017 (KS/17, clade 3C.3a1), and A/New Mexico/14/2018 (NM/18, clade 3C.2a2), were included for comparison.

**TABLE 1:** Antigenic characterization of hCK-derived H3N2 isolates using HAI assays

	Virus^*a*^	Ferret sera			
Clade		HK/14	SGP/16	KS/17	NM/18
3C.2a	HK/14	**640^*b*^**	160	20	80
3C.2a1	SGP/16	80	**320**	20	40
3C.3a1	KS/17	20	10	**640**	160
3C.2a2	NM/18	160	40	160	**640**
3C.2a1	48-hCK	80	40	40	40
	62-hCK	80	80	40	80
	102-hCK	40	20	20	40
	6-hCK	80	80	160	80
	8-hCK	160	80	80	80
	12-hCK	160	160	80	80
	15-hCK	160	160	80	80
	25-hCK	80	80	40	80
	42-hCK	160	320	160	160
	49-hCK	80	20	40	80
	65-hCK	40	40	40	40
	66-hCK	40	<20	<20	20
	67-hCK	20	<20	<20	20
	68-hCK	80	80	40	80
	76-hCK	80	80	20	20
	80-hCK	40	<20	<20	40
	144-hCK	80	80	80	160
	157-hCK	320	160	80	160
	258-hCK	80	40	40	40
3C.2a2	2-hCK	80	40	<20	80
	28-hCK	80	40	40	40
	35-hCK	160	80	80	160
	36-hCK	160	160	80	160
3C.2a3	205-hCK	40	<20	160	80

^*a*^ The isolates are denoted as *clinical sample identifier*-hCK.

^*b*^ Homologous titers are shown in bold, and the titers represent the mean values of duplicate measurements in the HAI assay.

**Fig 4:**
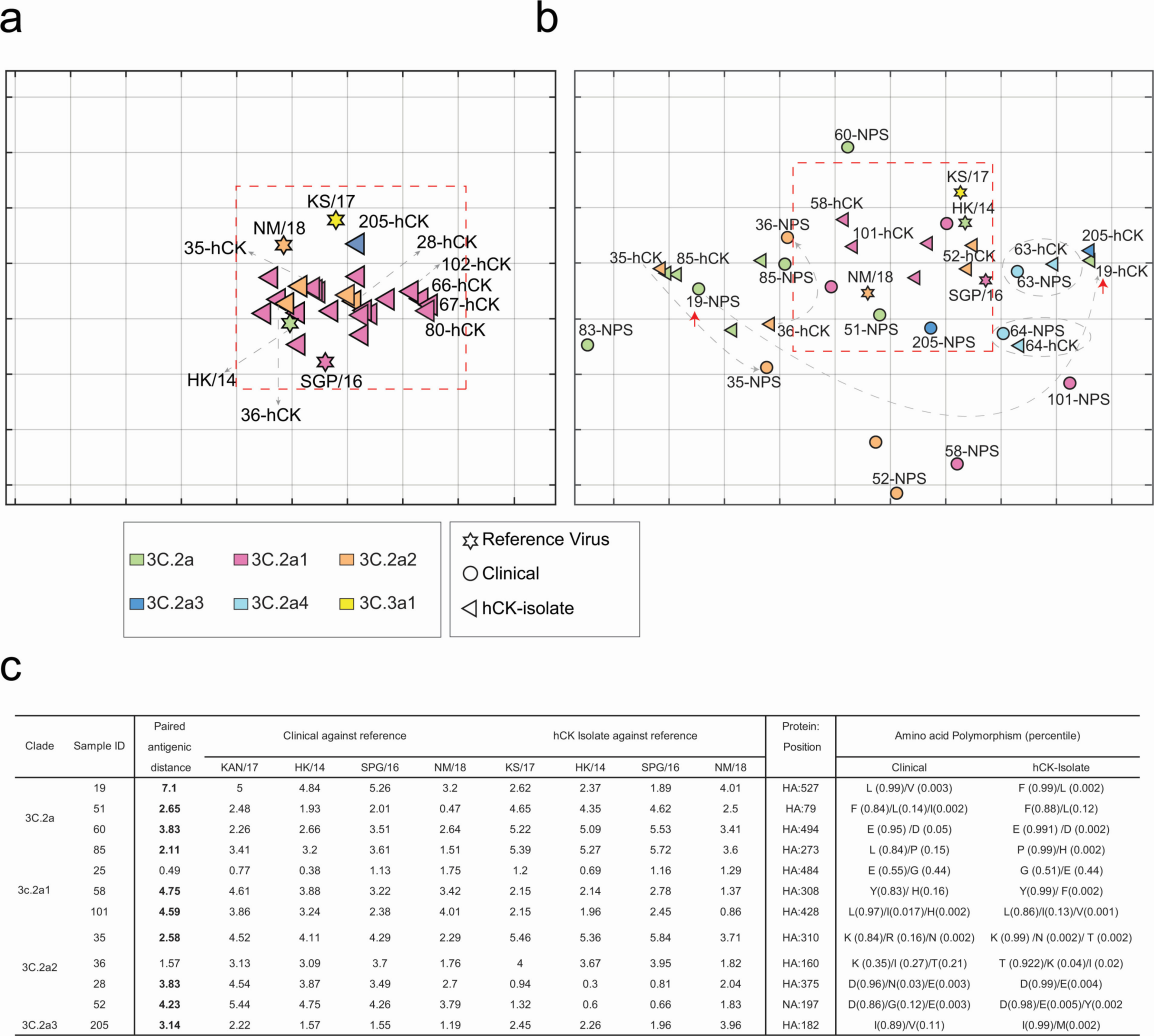
Intrahost polymorphisms shape antigenic diversity in H3N2 viruses. (**a**) HAI-based antigenic map of hCK-derived isolates tested against a panel of homologous reference antisera. (**b**) Polyclonal antibody-based polyPLA cartography of matched clinical specimens (circle) and hCK-derived isolates (triangles) tested against the same panel of reference antisera. Antigenic cartography was constructed by using http://sysbio.missouri.edu/AntigenMap (37, 38), which employed a low-reactor cutoff of 0.5 for polyPLA and 1:10 for HAI titer. A single unit on the antigenic map represents a log2 unit in each assay (39). The paired clinical samples and isolates are denoted as *clinical sample identifier*-NPS and *clinical sample identifier*-hCK, respectively. Reference viruses included HK/14 (clade 3C.2a; H3N2 component of the 2017–2018 Northern Hemisphere vaccines), SGP/16 (3C.2a1; H3N2 component of the 2018–2019 Northern Hemisphere vaccines), KS/17 (3C.3a1), and NM/18 (Clade 3C.2a2). Clade assignments are indicated by color-coded symbols. (**c**) Antigenic distances derived from polyPLA analysis for matched clinical specimens and corresponding hCK-derived isolates. The table summarizes antigenic coordinates and polyPLA distances relative to the reference strain, along with the associated amino acid polymorphisms identified in each sample, highlighting sequence variations contributing to observed antigenic differences.

HAI-based antigenic cartography showed that, as expected, the four reference strains were well separated, with HK/14 and SGP/16 being more antigenically similar (Fig. 4a), a pattern consistent with the polyPLA results (Fig. 4b). The majority of isolates clustered with their respective genetic clade reference strains in the HAI map, indicating close antigenic proximity. A few exceptions were observed, where isolates displayed >1.5 unit changes relative to their reference strains (e.g., 66-hCK and 67-hCK vs. SGP/16) (Fig. 4a).

In contrast, polyPLA-based antigenic cartography revealed greater heterogeneity, showing a broader spectrum of antigenic variation among the clinical samples (Table 2) (Fig. 4b). Notably, several clinical samples and their paired isolates exhibited pronounced antigenic differences. Among the 16 tested pairs, only five showed antigenic distances <2 units (each unit corresponding to a twofold change in polyPLA, with 1.12 polyPLA units equivalent to 1 HAI unit [39]), whereas the remaining pairs ranged from 2.1 to 7.1 units, indicating distinct antigenic properties (Fig. 4c). Overall, clinical samples showed greater antigenic divergence from their clade reference viruses than did the corresponding MDCK-derived isolates.

**TABLE 2:** Antigenic characterization of H3N2 viruses from clinical samples and their paired hCK-derived isolates using polyPLA

	Virus^*a*^	Ferret sera			
Clade		HK/14	SGP/16	KS/17	NM/18
3C.2a	HK/14	**5.40^*b*^**	4.79	8.50	3.28
3C.2a1	SGP/16	5.63	**5.92**	6.23	1.92
3C.3a1	KS/17	3.43	3.50	**10.16**	4.71
3C.2a2	NM/18	6.35	8.38	9.19	7.**12**
3C.2a	19-NPS	11.35	11.04	14.00	11.10
	19-hCK	0.66	2.81	4.40	2.02
	51-NPS	9.38	7.93	7.80	4.88
	51-hCK	9.75	11.56	11.43	11.33
	60-NPS	7.04	3.42	15.29	6.15
	60-hCK	9.69	11.74	14.96	12.47
	83-NPS	13.15	16.37	14.70	13.65
	83-hCK	7.49	9.88	13.10	9.82
	85-NPS	8.87	10.07	11.96	7.78
	85-hCK	10.65	11.44	15.26	12.52
3C.2a1	8-NPS	9.98	6.82	9.74	8.03
	8-hCK	8.16	4.41	7.37	5.10
	25-NPS	6.44	2.18	9.10	5.47
	25-hCK	7.87	4.12	8.77	4.25
	58-NPS	9.08	6.09	0.10	8.59
	58-hCK	7.46	5.93	12.52	6.81
	101-NPS	3.41	3.01	0.10	6.10
	101-hCK	6.78	6.70	11.22	7.34
3C.2a2	35-NPS	11.82	6.46	8.90	14.20
	35-hCK	11.53	11.11	15.55	12.20
	36-NPS	10.46	7.04	13.35	7.39
	36-hCK	9.06	10.21	10.63	10.56
	28-NPS	8.38	11.03	3.08	8.84
	28-hCK	5.16	3.06	7.79	4.79
	52-NPS	11.32	9.37	0.10	9.08
	52-hCK	6.20	3.73	6.99	4.59
3C.2a3	205-NPS	6.61	5.25	6.50	7.83
	205-hCK	0.21	0.95	4.76	4.42
3C.2a4	63-NPS	4.11	6.46	5.85	0.10
	63-hCK	0.71	3.06	5.30	4.14
	64-NPS	3.04	3.38	4.36	7.74
	64-hCK	1.36	7.49	3.47	4.52

^*a*^ The paired clinical samples and isolates are denoted as *clinical sample identifier*-NPS and *clinical sample identifier*-hCK, respectively.

^*b*^ Homologous titers are shown in bold, and the titers represent the mean values of triplicate measurements in the polyPLA assay.

Molecular analyses indicated that these antigenic differences were primarily associated with sequence variation in HA and NA. For instance, clinical sample 36-NPS exhibited a 1.6-unit antigenic distance from its paired isolate 36-hCK; the clinical sample contained a mixed HA:160K/I/T population at antibody-binding site B, with HA:160T predominating in the isolate 36-hCK. Similarly, clinical sample 19-NPS showed a 7.1-unit distance from isolate 19-hCK, accompanied by multiple polymorphisms. Within the HA gene, extensive intrahost quasispecies diversity was also detected at residues outside the five major canonical antibody-binding sites (40) (Fig. 4c). Nevertheless, sample 83-NPS displayed an antigenic distance of 3.6 units despite having no detectable polymorphisms in HA or NA but instead harbored several mutations in internal genes (Table S1). In contrast, samples 63-NPS and 64-NPS exhibited antigenic distances of <1 unit and showed no associated polymorphisms, consistent with their antigenic similarity.

Together, these findings demonstrate that virus isolation in MDCK cells alters variant composition in genetically diverse clinical populations, reducing intrahost diversity and leading to measurable antigenic changes. Together, our findings highlight the need for direct antigenic and genetic profiling of clinical specimens to better capture viral diversity and inform vaccine strain selection.

### Antigenic variations driven by quasispecies among clinical samples

We hypothesized that intrahost polymorphisms within clinical samples generate a continuum of antigenic phenotypes that are not captured by consensus sequences. To test this, we focused on residue 160 of the HA protein, located within antibody-binding site B, a residue where HA:160T modulates antibody recognition through N-linked glycosylation (17) and exhibited the highest entropy in our data set (Fig. 2c). Using reverse genetics (PR8 + 2 system), we generated three recombinant viruses, each carrying one naturally occurring variant at this position (HA:160K, HA:160T, or HA:160I). To mimic the quasispecies composition observed in clinical samples, we created 11 viral populations: three pure variants and eight defined mixtures, including one replicating the composition of clinical sample 36-NPS (rg160K: rg160T:rg160I; 35:27:21).

**Fig 5:**
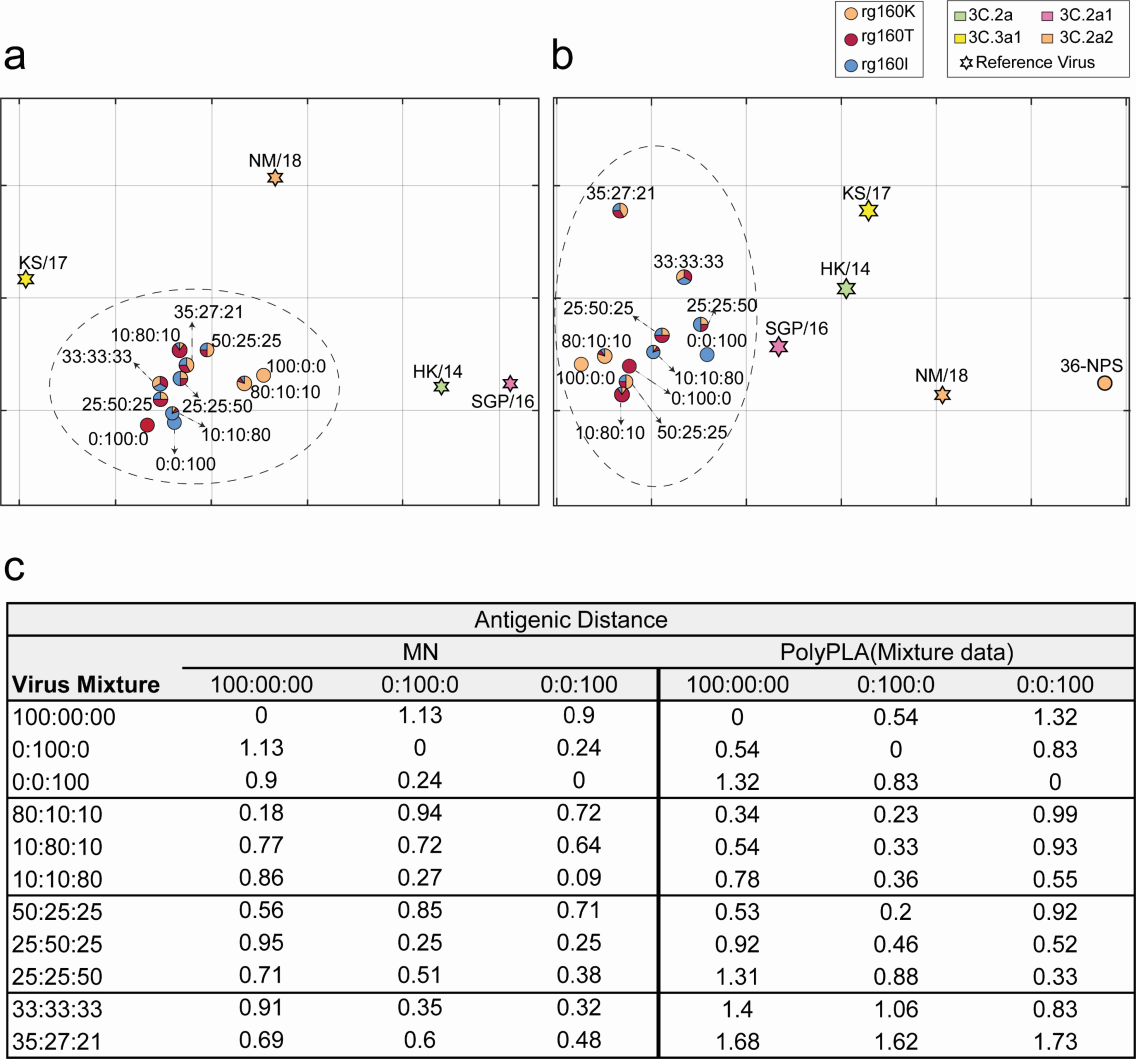
Antigenic variations driven by quasispecies among clinical samples. (**a**) Antigenic cartography based on MN data with rg160K, rg160T, and rg160I, as well as engineered mixtures at defined ratios (rg160K:rg160T:rg160I). (**b**) Antigenic cartography based on polyPLA data for the same virus mixtures. Antigenic cartography was constructed by using http://sysbio.missouri.edu/AntigenMap (37, 38), which employed a low-reactor cutoff of 0.5 for polyPLA and 1:20 for MN titer. A single unit on the antigenic map represents a log2 unit in each assay (39). The paired clinical samples and isolates are denoted as *clinical sample identifier*-NPS and *clinical sample identifier*-hCK, respectively. Reference viruses included HK/14 (clade 3C.2a), SGP/16 (3C.2a1), KS/17 (3C.3a1), and NM/18 (clade 3C.2a2). Clade assignments are indicated by color-coded symbols. (**c**) Antigenic distances derived from MN and polyPLA data relative to each mixture ratio (rg160K:rg160T:rg160I), illustrating quantitative antigenic shifts associated with varying proportions of viral variants in the population.

Because recombinant viruses had low HA titers, antigenic profiling was performed using MN and polyPLA assays. Variation at residue HA:160 altered antigenic phenotypes by approximately 1–2 units (Fig. 5). Pure variants occupied distinct positions in antigenic space, whereas mixed populations displayed intermediate or displaced locations depending on the dominant residue. However, their antigenic properties did not follow simple proportional averaging. For instance, rgT160-enriched mixtures (10:80:10) and (50:25:25) clustered near the rgT160 (0:100:0) virus, showing incremental antigenic distances of 0.18 and 0.56 units, respectively, corresponding to decreasing proportion of rgT160. In contrast, rgK160-enriched mixtures (10:80:10) and (25:50:25) were displaced by 0.72 and 0.25 antigenic units, respectively, relative to the rgK160 (100:0:0) virus in the MN-derived map (Fig. 5a). Although MN and polyPLA assays differed slightly in the exact antigenic positions, both revealed the same overall trends. The 35:27:21 (rg160K: rg160T:rg160I) mixture showed an antigenic pattern similar to that of the corresponding clinical isolate, albeit with smaller changes,

Detailed MN titers confirmed non-additive antigenic effects of residue 160 polymorphisms (Table 3). The HA:160K virus was most sensitive to neutralization, particularly by HK/14 antisera (1:640), whereas HA:160T (1:160) and HA:160I (1:320) variants showed reduced and intermediate titers, respectively. Mixed populations produced a continuum of titers (1:160–1:640) that did not scale linearly with variant composition. For example, mixtures containing 50% T160, with the remainder composed of K160 or I160, maintained high titers (1:640) against HK/14 antisera but dropped to 1:160 when the T160 ratio fell to 33% or lower. PolyPLA analyses mirrored these patterns (Table 3).

**TABLE 3:** Antigenic characterization of mixtures of rg160K:rg160T:rg160I using MN and polyPLA assays^*a*^

Clade	Virus	Microneutralization (ferret sera)				PolyPLA (ferret sera)			
		HK/14	SGP/16	KS/17	NM/18	HK/14	SGP/16	KS/17	NM/18
3C.2a	HK/14	**1,920**	2,560	20	320	**5.4**	4.79	8.50	3.28
3C.2a1	SGP/16	5,120	**5,120**	20	240	5.63	**5.92**	6.23	1.92
3C.3a1	KS/17	30	20	**160**	160	3.43	3.50	**10.16**	4.71
3C.2a2	NM/18	800	320	320	**3,840**	6.35	8.38	9.19	**7.12**
3C.2a2	100:0:0	640	320	80	320	3.414	3.948	0.945	3.177
	0:100:0	160	80	40	160	4.719	3.610	2.062	3.297
	0:0:100	320	80	40	160	4.307	4.862	4.166	4.590
	80:10:10	640	160	40	320	3.573	4.356	1.889	3.018
	10:80:10	320	80	40	320	4.108	4.886	1.652	3.892
	10:10:80	320	80	40	80	3.589	4.597	2.998	3.868
	50:25:25	640	80	40	160	4.661	4.500	1.837	2.917
	25:50:25	160	80	40	160	4.134	3.891	3.389	3.325
	25:25:50	320	80	40	160	3.167	4.942	4.523	4.313
	33:33:33	160	80	40	160	3.273	4.319	4.774	3.136
	35:27:21	320	80	40	160	1.723	1.000	4.237	2.796

^*a*^ Bold values denote homologous virus–reference comparisons, where each virus is evaluated against its corresponding reference strain in the microneutralization and polyPLA assays. The diagonal entries represent self-matched interactions and serve as internal benchmarks for maximal reactivity within each assay.

Together, these findings demonstrate that polymorphisms at residue HA:160 reshape antigenic recognition in a non-additive manner. Mixed quasispecies exhibit emergent antigenic profiles distinct from clonal viruses, underscoring how intrahost diversity contributes to influenza antigenic complexity.

## DISCUSSION

This study provides direct evidence that intrahost polymorphisms in clinical influenza A(H3N2) samples contribute to measurable antigenic diversity and that virus propagation in MDCK cells imposes strong purifying selection that alters both genetic and antigenic properties. By combining deep sequencing and serological assays, including a high-sensitivity proximity ligation approach polyPLA (33, 34), we demonstrate that clinical specimens harbor substantial within-host variation, particularly at surface-exposed HA residues. Virus isolation in MDCK cells markedly reduces this diversity through selective enrichment of adaptive variants, and polymorphisms at key sites such as HA:160 yield non-linear antigenic outcomes that cannot be predicted from consensus sequences alone. Together, these results refine our understanding of how intrahost genetic evolution, culture adaptation, and assay methodology influence influenza antigenic characterization and vaccine strain selection (11, 30, 41).

Our findings show that propagation of human A(H3N2) viruses in MDCK-derived cells markedly reshapes intrahost diversity (Fig. 2 and 3) and compresses antigenic profiles relative to those in clinical specimens (Fig. 4 and 5). Such culture-induced narrowing represents a distinct source of bias in current GISRS workflows, which rely primarily on passaged isolates for antigenic characterization. While this effect acts in parallel with other contributors to vaccine mismatch, such as ongoing antigenic evolution and pre-existing immunity (14), it nonetheless selectively enriches specific HA variants and obscures the broader antigenic landscape present *in vivo*. These results support incorporating approaches that directly assess antigenicity from uncultured clinical material to more accurately capture the diversity circulating in human infections.

The pronounced reduction in genetic diversity following virus propagation in MDCK cells reflects strong purifying selection, likely driven by both biological and experimental factors. In natural human infections, viral populations are shaped by heterogeneous humoral immune pressures, including pre-existing antibodies and mucosal immunity, which sustain multiple coexisting variants within a host (42, 43). This complex adaptive immune environment promotes the emergence of intrahost polymorphisms, particularly at immunodominant epitopes such as the HA antibody-binding sites (44). In contrast, MDCK cells provide a more uniform environment without immune selection derived from adaptive immunity. Under these conditions, viral fitness depends primarily on receptor binding compatibility, cell entry, and replication efficiency. Variants that replicate more efficiently in canine cells, such as those with altered glycosylation or receptor-binding properties, are preferentially amplified, while others are diminished through a population bottleneck. Similar cell-adaptive mutations in influenza HA have been reported in serial passage studies (45). Non-immunologic factors, including differences in sialylated receptor distribution or host restriction factors, may further shape this selection pressure during virus propagation (46, 47). Because HA and NA are under strong structural and functional constraints, culture conditions may tend to favor variants that maximize replication or stability rather than antigenic diversity. Importantly, because these adaptive changes occur during the very isolation processes used for vaccine candidate evaluation, they may systematically bias antigenic data that inform vaccine strain selection.

Our experimental reconstruction of quasispecies mixtures revealed that polymorphisms at a single site, HA:160, can produce non-linear antigenic effects (Fig. 5). Although mixtures tended to drift toward the dominant variant in antigenic space, the magnitude of these changes was disproportionate to the variant ratios, indicating non-additive relationships between sequence composition and antigenic phenotype. This emergent behavior likely reflects epistatic and structural interactions within the HA trimer, where coassembly of monomers bearing different residues and alterations in glycosylation modulate antibody binding in ways not predictable from linear averaging (19, 48). On the other hand, these results may suggest a distinction between genetic-level and protein-level variations. Nucleotide polymorphisms do not necessarily translate to equivalent antigenic changes, as the antigenic phenotype is shaped by amino acid context, glycan occupancy, and conformational flexibility (14). Future work should incorporate glycoproteomic and structural analyses to determine how intrahost sequence heterogeneity influences site-specific glycosylation and epitope accessibility at the protein level.

Although HA exhibited the strongest evidence of purifying selection, reductions in genetic diversity were also observed in NA and several internal segments (Table S1). Within HA, selection extended beyond the receptor-binding domain into the HA2 stalk and transmembrane regions, suggesting that adaptation to MDCK cells affects structural domains important for membrane fusion, virion stability, or assembly. Similar adaptation patterns have been reported for influenza viruses propagated in eggs and other cell lines (27). The observation that purifying selection acts across multiple gene segments suggests that culture adaptation is multigenic, with effects on polymerase activity, packaging, and virus release (49). These findings underscore the importance of minimizing *in vitro* passage when assessing antigenic properties for vaccine strain selection.

PolyPLA proved to be a powerful tool for assessing antigenic relationships directly from clinical materials, capturing a broader range of antibody–antigen interactions than conventional HAI or MN assays (34, 39). Because polyPLA measures total antibody binding rather than only neutralization, it detects both neutralizing and non-neutralizing antibody interactions, including those directed against the HA stalk and NA (50). The greater antigenic diversity observed by polyPLA compared to MN likely reflects its sensitivity to subtle conformational or glycan-mediated differences that may not fully impair neutralization but still modify antibody binding. From a surveillance perspective, these properties make polyPLA especially valuable for detecting fine-scale antigenic heterogeneity in uncultured specimens, although interpretation must consider that not all detected antibodies confer protection. The combined use of polyPLA with neutralization assays could thus provide a more complete understanding of antigenic evolution and immune escape in circulating influenza viruses.

The number of clinical–isolate pairs examined in this study was limited and restricted to A(H3N2) in a single influenza season. Nevertheless, the overall patterns were consistent, revealing strong purifying selection during virus propagation and its influence on antigenic diversity. Although similar trends were not observed across all viral lineages, these results provide a mechanistic basis for understanding how culture adaptation can distort the antigenic landscape used to guide vaccine strain selection. Expanding this approach to a broader range of clinical samples, influenza subtypes, such as A(H1N1)pdm09 and zoonotic viruses infecting humans, and propagation systems (e.g., beyond hCK) will be essential to determine the generality of these mechanisms and their impact on surveillance data. Moreover, the *in vivo* consequences of the identified antigenic and genetic changes, including their effects on transmissibility, immune escape, and ultimately vaccine performance, remain to be determined. Together, these directions will extend the current findings beyond *in vitro* characterization, advancing efforts to improve the accuracy of antigenic analysis and the reliability of vaccine strain selection.

The experimental workflow used here aligns with current GISRS practices, in which antigenic characterization relies primarily on passaged isolates tested by HAI and, increasingly, MN assays (14). While alternative MDCK derivatives, including hCK cells, can facilitate isolation of contemporary H3N2 viruses, our data indicate that culture-based propagation continues to impose selective bottlenecks regardless of the substrate. In contrast, polyPLA enables direct antigenic assessment from uncultured clinical material, capturing antigenic relationships that may be obscured after isolation and passage (34, 39). We therefore emphasize polyPLA not as a replacement for existing GISRS assays but as a complementary, high-resolution approach that can contextualize isolate-derived data, especially when fine-scale antigenic heterogeneity or culture-associated adaptation is of concern.

In summary, our findings demonstrate that influenza viruses circulate as dynamic, heterogeneous populations within infected hosts, and that culture-based isolation imposes selective bottlenecks that distort this natural diversity. Intrahost polymorphisms at antigenically important residues can generate emergent, non-linear antigenic behaviors that challenge consensus-based interpretations of antigenic evolution. Direct characterization of viruses from clinical specimens, integrating serological, genomic, and structural analyses, provides a more faithful representation of the antigenic landscape of circulating strains. Such approaches will be critical for improving the fidelity of antigenic surveillance and enhancing the precision of influenza vaccine strain selection.

## MATERIALS AND METHODS

### Clinical samples

NPS specimens were collected from patients with influenza-like illness during the 2017–2018 influenza season as part of influenza surveillance conducted by the Mississippi Department of Public Health. These samples were de-identified prior to laboratory analysis. All samples were stored at −80°C.

### Cells

Humanized MDCK cells (hCK), which overexpress α-2,6-sialoglycans and almost completely delete the expression of α-2,3-sialoglycans (51), were kindly provided by Dr. Yoshihiro Kawaoka from the University of Wisconsin-Madison, USA. The MDCK-Siat1 cells, which overexpress α-2,6-sialyltransferase but show a marginal decrease in α-2,3-sialylation (35), were kindly provided by Dr. Mikhail Matrosovich from Philipps University, Germany. The MDCK-CCL34 cells were obtained from the Biodefense and Emerging Infections Research Resources Repository (ATCC Cat. No. CCL-34). MDCK-CCL34 cells express relatively low levels of both α-2,3- and α−2,6-sialic acids with slightly higher levels of α-2,3-sialylation compared with α-2,6-sialylation (52). All cells were maintained at 37°C under 5% CO_2_ in Dulbecco’s modified Eagle medium (Gibco DMEM; Thermo Fisher Scientific, Waltham, MA, USA; Cat. No. 11965-084) supplemented with 10% fetal bovine serum (Gibco; Thermo Fisher Scientific Baltics, Vilnius, Lithuania; Cat. No. A52568-01).

### Viruses and ferret sera

A/New Mexico/14/2018 (H3N2) (NM/18, Clade 3C.2a2), A/Hong Kong/4810/2014(H3N2) (HK/14, Clade 3C.2a), A/Singapore/Inlimh-16-0019/2016 (H3N2) (SGP/16, Clade 3C.2a1), and A/Kansas/14/2017 (KS/17, Clade 3C.3a1) viruses were obtained from the International Reagent Resource (https://www.internationalreagentresource.org/Home.aspx) and propagated in MDCK-Siat1 cells. Ferret sera were generated by intranasally infecting two 4- to 6-month-old adult ferrets with each strain, and sera were collected four weeks post-infection, pooled, and used for serological analyses.

### Virus isolation

The clinical isolates were diluted at least 1:100 and inoculated into MDCK-hCK cells and MDCK-SIAT1 following the virus isolation protocols described in the WHO *Global Influenza Surveillance Network Manual for the Laboratory Diagnosis and Virological Surveillance of Influenza* (53).

### Nucleic acid extraction

Viral RNA was extracted from NPS specimens using the Applied Biosystems MagMAX Viral/Pathogen Nucleic Acid Isolation Kit (Cat. No. A48310) following the manufacturer’s protocol. The extraction was automated using the Thermo Fisher KingFisher Flex Purification System, which employs magnetic bead-based nucleic acid isolation to ensure high-quality and consistent RNA recovery. The extracted RNA was eluted in (90 µL) RNase-free water or buffer and stored at −80°C until further analysis.

### Quantitative RT-PCR

A real-time quantitative RT-PCR (qRT-PCR) assay targeting the conserved matrix (M) gene of IAV was performed. The primer and probe sequences were adapted from the WHO influenza diagnostic protocols (53), and the assay was performed using the QuantStudio 6 Real-Time PCR System (Applied Biosystems, Thermo Fisher Scientific). Positive and negative controls were included in each run, with IAV RNA serving as the positive control and nuclease-free water or RNA from non-infected cells as the negative control. Samples with cycle threshold (Ct) values ≤ 37.5 were determined to be positive.

### Recombinant virus generation using reverse genetics

All recombinant viruses were generated using the eight-plasmid influenza reverse genetics system. The HA and NA gene segments from clinical sample 36-NPS were synthesized and cloned into the pHW2000 vector (GeneUniversal, Newark, DE). Three HA constructs, each encoding one of the naturally occurring variants at residue 160 (K, T, or I), were used to generate recombinant viruses. Three 6:2 reassortant viruses, designated rg160K, rg160T, and rg160I, were rescued using the HA and NA from sample 36-NPS combined with the six internal gene segments of A/Puerto Rico/8/1934 (H1N1) (PR8). To enhance virus rescue efficiency, the noncoding regions of both the HA and NA genes were replaced with those from PR8. In addition, the HA N-terminal signal peptide, C-terminal transmembrane domain, and cytoplasmic tail were substituted with corresponding PR8 sequences.

Virus rescue was performed by co-transfecting HEK 293T and hCK (6:1 ratio) seeded at 70% confluence in six-well plates with 1 µg of each of the eight plasmids (8 µg total DNA) using 16 µL of TransIT-LT1 transfection reagent (Mirus Bio, Madison, WI; Cat. No. MIR 2305). After 6 h, the transfection medium was replaced with 2 mL of Opti-MEM (Thermo Fisher Scientific; Cat. No. 11058021). At 24 h post-transfection, 1 mL of Opti-MEM containing 1 µg/mL TPCK-treated trypsin (Sigma-Aldrich; Cat. No. T1426-250MG) was added. Supernatants were collected 72 h post-transfection and inoculated into hCKat 90% confluence in T75 flasks for virus amplification. Cultures were harvested when >70% cytopathic effect (CPE) was observed; if CPE was <70% at 72 h post-infection, supernatants were collected at that time point. Recovered viral genomes were verified to confirm the presence of the intended mutations and the absence of undesired substitutions. Viral titers were determined in hCK by TCID₅₀ assay as described above.

### HA and HAI assays

HA and HAI assays were performed by using 0.75% guinea pig erythrocytes as described by the WHO Global Influenza Surveillance Network Manual for the Laboratory Diagnosis and Virological Surveillance of Influenza (53). Guinea pig erythrocytes were obtained from Lampire Biological Products (Everett, PA). The guinea pig erythrocytes were washed three times with 1 × PBS (pH 7.2) before use and then diluted to 0.75% in 1 × PBS (pH 7.2).

### PolyPLA assay

PolyPLA assays were performed as previously described (34, 39). Briefly, polyclonal ferret sera against reference vaccine strains and NP-specific monoclonal mouse antibodies (BEI Resources, Cat. No. NR-43899) were purified using Protein A/G cartridges (Thermo Fisher, Cat. No. 89930) and biotinylated with the Biotin-XX Microscale Protein Labeling Kit (Invitrogen, Cat. No. B30010). Excess biotin was removed by dialysis using Slide-A-Lyzer mini dialysis cassettes (Thermo Fisher Scientific, Cat. No. 69572) against PBS at 4°C. For proximity probe generation, biotinylated antibodies were incubated with 3′ and 5′ TaqMan Prox-Oligos (Thermo Fisher Scientific, Cat. No. 4448549) to generate proximity probes A and B. For PLA reactions, virus samples (clinical specimens or isolates) were incubated with an equal mixture of probes A and B at 4°C overnight. Ligation and protease treatments were carried out according to the manufacturer’s instructions for the TaqMan Protein Assay protocol. Quantitative PCR detection was performed using TaqMan Fast Universal PCR Master Mix (Thermo Fisher Scientific, Cat. No. 4448616) and standard cycling conditions on a QuantStudio Real-Time PCR System.

The NPC was used as a reference background, and the threshold cycle (Ct) value given dictated the non-target ligation signal noise of the assay. A total of three replicates for each sample and control were performed. To calculate the ΔCt values: Average Ct (NPC) – Average Ct (sample), which represented the true target-mediated signal above background. The cutoff of ΔCt ≥ 3.00 was used for qualitative analysis of viral and antibody binding, according to the Taqman Protein Assays Sample Prep and Protocol (54).

To compare the antigenic properties across the testing antigens (viruses), we computed the polyPLA unit between antigen (virus) and antibody using the following equation: polyPLA = *a* × (polyΔCt−monoΔCt) + *b*. To improve our computation, we simply used *a* = 1.00 and *b* = 10.00. The *b* = 10.00 enabled us to avoid negative numbers. If monoΔCt < 3.00, polyPLA will be assigned as “<0,” meaning that the viral loads were too low for analysis. polyPLA is sensitive in detecting viral loads of approximately 10^3^ TCID_50_/mL (39).

### MN assay

The MN assay was performed as described in WHO influenza diagnostic protocols (53). Briefly, the tested ferret sera were treated with Receptor Destroying Enzyme (Hardy Diagnostics, Cat. No. 370013) overnight, then heat-inactivated at 56°C for 30 min prior to use. Two-fold serial dilutions of each serum sample were prepared in serum-free MEM supplemented with 1% penicillin–streptomycin (Thermo Scientific, Cat. No. 15140122). An equal volume containing 100 TCID_50_ of the tested influenza virus was added to each dilution, and an additional well without sera as virus control (VC); incubated at 37°C for 1 h to allow antibody–virus binding. 1.5 × 10^4^ hCK cells per well were added to the serum–virus mixtures, and an additional well with virus or sera as cell control (CC) in 96-well plates and was incubated at 37°C with 5% CO₂. After 18–20 h, viral replication was quantified by ELISA using an anti-influenza nucleoprotein rabbit monoclonal antibody (BEI Resources, Cat. No. NR-43899), followed by HRP-conjugated anti-rabbit secondary antibody (Thermo Fisher Scientific, Cat. No. A16110) and TMB substrate (Thermo Scientific, Cat. No. 34029) detection. Neutralizing antibody titers were defined as the reciprocal of the highest serum dilution that completely inhibited viral replication. Determine the virus neutralization antibody 50% titer of each serum using the following equation: *x* = [(average OD of VC wells) + (average OD of CC wells)]/2, where *x* is the OD value at which 50% of the MDCK cells were infected. All values below or equal to *x* are positive for neutralization activity. The reciprocal serum dilution corresponding to that well is the 50% neutralization antibody titer for that serum sample.

### NGS and data analyses

Two-step RT-PCR amplification with influenza A universal primers was used to amplify full genome sequences (55, 56). Sequencing libraries were prepared using the Illumina Nextera DNA Flex Library Prep Kit and sequenced on NextSeq 500/550 System using its High Output Reagent Kit v2 (300 Cycles). Raw reads were processed using BBDuk from the BBMap v39.01 package (https://sourceforge.net/projects/bbmap/). Adapter sequences were removed by trimming the terminal 30 bases using a k-mer size of 17 with a maximum Hamming distance of 1 to ensure accurate adapter removal. Reads with an average Phred quality score <20 were excluded, corresponding to a 1% error probability for improved fidelity in low-frequency variant detection. Trimmed reads were assembled using the CDC Iterative Refinement Meta-Assembler (IRMA) Flu module (57). In accordance with validated influenza-specific IRMA parameters, we applied a minimum per-base quality score threshold of 20 during read gathering and alignment (Phred quality score of 20). Intrahost single-nucleotide variants (iSNVs) were required to have a per-site depth of ≥100 reads, at least two supporting reads, an allele frequency ≥0.8%, an average variant-base quality ≥Q24. We ensured that all samples had sufficient depth, with median coverage well above 5,000×. These thresholds reflect IRMA’s empirically benchmarked settings for influenza quasispecies reconstruction and balance sensitivity for detecting intrahost variants (58) with protection against platform-specific sequencing noise. IRMA output files (consensus FASTA sequences and segment-level BAMs) were parsed with a custom Python script, which extracted the segment-wise consensus sequences and concatenated them into complete viral genomes. Each gene segment was aligned using the ClustalW method in BioEdit v7.2.6.1 (59), with the A/Switzerland/9715293/2013 (H3N2) strain serving as the reference.

### Genetic diversity

Nucleotide and amino acid substitutions between the clinical and isolate-derived sequences were identified through multi-sequence alignment. BAM files produced by the IRMA pipeline were further processed using Samtools v1.13, and reads were realigned to the reference genome using BWA-MEM to produce standardized SAM files. iSNVs were identified by IRMA using its validated influenza-specific pipeline. Amino-acid-level polymorphisms were assessed using DiversiTools (https://github.com/josephhughes/DiversiTools), which extracts the nucleotide frequencies directly from the IRMA-generated BAM files and converts them into an amino-acid frequency profile. To ensure that detected polymorphisms represented true biological variation, we required a minimum amino acid coverage of ≥100 reads per position. This approach facilitated the comparison of amino acid composition at key positions between clinical samples and cell-culture isolates. At each amino acid position, the proportional abundance of the top, secondary, and tertiary residues was calculated, allowing direct comparison of amino acid composition between clinical specimens and their corresponding cell-culture isolates. This approach helps to identify changes in variant frequencies, such as the loss of minority residues or the fixation of a single dominant amino acid, which often occurs during virus culture. The distribution of these variant proportions was visualized alongside Shannon entropy values to capture both the magnitude and complexity of intrahost polymorphism. These analyses provided detailed insight into how MDCK cell culture shapes H3N2 viral diversity and selects for adaptive mutations.

### Shannon entropy

Genetic diversity was quantified using Shannon entropy based on allele frequencies at variable sites. The entropy *H*(*x*) was calculated as $H\left(x\right)=-\sum_{k=0}^{n}P(K)\log_{2}P(K)$, where *P*(*k*) represents the proportion of the amino acid at position *k*. Positions with higher variability in amino acids showed greater entropy, indicating higher polymorphism levels.

### Phylogenetic analyses

To investigate the evolutionary relationships of H3N2 IAVs, time-scaled phylogenetic analyses were performed for all eight gene segments using both clinical specimens and their corresponding isolates. In addition, 30 representative H3N2 strains circulating up to 2018 were retrieved from the GISAID EpiFlu database (http://www.gisaid.org), incorporated to capture representative clade diversity. Bayesian phylogenetic inference was conducted using BEAST v1.10.5(57) under the HKY substitution model with four gamma rate categories, an uncorrelated relaxed molecular clock, and a coalescent exponential growth prior. Markov chain Monte Carlo chains were run for 20 million iterations with a 10%–15% burn-in, and convergence was assessed in Tracer v1.7.2, applying a minimum effective sample size (ESS) threshold of 200. MCC trees were summarized with TreeAnnotator v1.10.5, using median node heights and a posterior probability cutoff of 0.7. The resulting trees were rooted to the earliest lineage (A/Perth/16/2009) and visualized using the ggtree R package v4.5.1.

### Antigenic cartography

Antigenic cartography was constructed by using https://www.internationalreagentresource.org/Home.aspx (37, 38), which employed a low-reactor cutoff of 0.5 for polyPLA and 1:10 for HAI titer, and 1:20 for MN assay, and a low rank (rank = 3) matrix completion was used to minimize noise from the HAI data. A single unit on the antigenic map represents a log2 unit in each assay (39). To minimize noise in serological data and reflect antigenic distances embedded in the data, low-rank matrix completion and multiple-dimensional scaling were utilized to generate the map.

### Structural visualization

Structural analyses were performed in PyMOL using the crystal structure 4WE8 as the template for visualization.

### Statistical analyses

Polymorphism analysis was conducted using the DiversiTools pipeline (https://josephhughes.github.io/DiversiTools/tutorial.html). Phylogenetic trees were generated and visualized using the *ggtree* package in R. Polymorphism and entropy plots were created using custom scripts in Python.

## Data Availability

All data associated with this study are available in GenBank under BioProject no. PRJNA1346101.

## SUPPLEMENTAL MATERIAL

Supplemental material jvi.01775-25-s0001.pdf Table S1; Fig. S1 and S2.

ASM does not own the copyrights to Supplemental Material that may be linked to, or accessed through, an article. The authors have granted ASM a non-exclusive, world-wide license to publish the Supplemental Material files. Please contact the corresponding author directly for reuse.
