# Supplementary material for: Intrahost HA polymorphisms and culture adaptation shape antigenic profiles of H3N2 influenza viruses
Source: J Virol. 2026 Jan 7;100(2):e01775-25. doi: 10.1128/jvi.01775-25 (PMC12831994; doi:10.1128/jvi.01775-25)
Supplement: Supplemental material — Table S1; Fig. S1 and S2. [file jvi.01775-25-s0001.pdf]

SUPPLEMNARY INFORMATION

Title: Intrahost HA polymorphisms and culture adaptation shape antigenic profiles of H3N2 influenza viruses

Kritika Prasai[1,2,3], Zunlin Yang[1,2,4], Minhui Guan[1,2,4], Tao Li[5], Daphne Ware[6], Jun Hang[5], Xiu-Feng Wan[1,2,3,4]

[1]NextGen Center for Influenza and Emerging Infectious Diseases, University of Missouri, Columbia, Missouri, USA;
[2]Department of Electrical Engineering & Computer Science, College of Engineering, University of Missouri, Columbia, Missouri, USA;
[3]Bond Life Sciences Center, University of Missouri, Columbia, Missouri, United States of America;
[4]Department of Molecular Microbiology and Immunology, School of Medicine, University of Missouri, Columbia, Missouri, USA;
[5]Viral Diseases Branch, Walter Reed Army Institute of Research, Silver Spring, Maryland, USA;
[6]Mississippi Public Health Laboratory, Jackson, MS 39216, USA.

* Correspondence: wanx@missouri.edu

List of Supplementary Tables and Figures

TABLE S1. Comparison of genetic diversity between clinical H3N2 samples and their paired hCK-derived isolates. Shannon entropy values and the number of polymorphic sites across HA, NA and all internal gene are shown for each matched pair. Predominant variants were indicated in bold, and variants shown in red denote changes observed in the isolates compared to the corresponding clinical samples. The polymorphisms and entropy values were analyzed using DiversiTools (https://github.com/josephhughes/DiversiTools).

FIGURE S1. Time-scaled Bayesian phylogenetic trees of HA (a), NA (b), and six internal gene segments PB2 (c), PB1 (d), PA (e), NP (d), M (f), and NS (g) from A(H3N2) collected during the 2017–2018 influenza season. Clinical samples are shown in red, and their corresponding hCK-derived isolates in blue; representative circulating H3N2 strains retrieved from the GISAID EpiFlu database (collected up to 2018) are shown in black for clade context. Phylogenetic trees were inferred using BEAST v1.10.5 under an HKY substitution model with a unrelated relaxed clock and a coalescent exponential growth prior. Posterior probabilities ≥0.70 are indicated at key nodes.

FIGURE S2. Comparison of intrahost genetic diversity between clinical H3N2 specimens and their matched hCK-derived isolates across internal gene segments. Shannon entropy values, derived from deep sequencing data, are plotted for each amino acid position of PB2 (a), PB1 (b), PA (c), NP (d), M (e), and NS (f). Red circles represent clinical samples, while blue triangles denote corresponding isolates. Higher entropy values reflect greater amino acid diversity within a sample. Across all segments, clinical specimens generally exhibited greater variability than their matched isolates, indicating that virus culture imposes purifying selection and reduces within-sample diversity. Entropy values were calculated from iSNVs identified using DiversiTools (https://github.com/josephhughes/DiversiTools).

TABLE S1. Comparison of genetic diversity between clinical H3N2 samples and their paired hCK-derived isolates. Shannon entropy values and the number of polymorphic sites across HA, NA and all internal gene are shown for each matched pair. Predominant variants were indicated in bold, and variants shown in red denote changes observed in the isolates compared to the corresponding clinical samples. The polymorphisms and entropy values were calculated using DiversiTools (https://github.com/josephhughes/DiversiTools).

Sample ID	Protein	Amino acid (percentile)			Entropy
		Position	Clinical	hCK isolate	Clinical/hCK isolate
18	HA	385	**F** (0.91) /I (0.08)	**I** (0.99)/S (0.004)	0.29/0.25
19	HA	527	**L** (0.99)/V (0.003)	**F** (0.99)/L (0.002)	0.04/0.05
25	HA	484	**E** (0.55)/G (0.44)	**G** (0.51)/E (0.44)	1.01/1.02
35	HA	310	**K** (0.84)/R (0.16)/N (0.002)	**K** (0.99) /N (0.002)/ T (0.002)	0.68/0.04
36	HA	158	**N** (0.96)/D(0.02)/I(0.003)	**N** (0.75)/D(0.24)/Y(0.0014)	0.23/0.82
36	HA	160	**K** (0.35)/I (0.27)/T(0.21)	**T** (0.92)/K (0.04)/I (0.02)	1.5/0.37
51	HA	79	**F** (0.84)/L(0.14)/I(0.002)	**F** (0.88)/L(0.12)/Y(0.002)	0.62/0.55
58	HA	308	**Y** (0.83)/ H(0.16)	**Y** (0.99)/ F(0.002)	0.66/0.05
58	HA	407	**Q** (0.88) /R (0.11) /H (0.002)	**Q** (0.99) /H (0.004) /L (0.002)	0.53/0.06
60	HA	494	**E** (0.95) /D (0.05) /V (0.002)	**E** (0.99) /D (0.002) /V (0.002)	0.29/0.05
85	HA	273	**L** (0.84)/P (0.15)	**P** (0.99)/H (0.002)	0.65/0.05
101	HA	428	**L** (0.97)/I(0.017)/H(0.002)	**L** (0.86)/I(0.13)/V(0.001)	0.14/0.57
102	HA	228	**S** (0.93)/T(0.06)/R(0.002)	**S** (0.99)/R(0.002)	0.37/0.05
102	HA	478	**I** (0.98) /M (0.016) /L (0.002)	**I** (0.73) /M (0.26) /L (0.002)	0.14/0.85
144	HA	92	**R** (0.98) /K (0.022) /S (0.001)	**R** (0.96) /K (0.03) /S (0.009)	0.17/0.27
157	HA	174	**N** (0.81) /K (0.10) /S (0.06)	**N** (0.98) /S (0.007) /K (0.006)	0.86/0.12
205	HA	225	**D** (0.88) /G (0.12)	**D** (0.99) /E (0.003) /Y (0.003)	0.53/0.06
205	HA	262	**N** (0.86) /S (0.14) /T (0.001)	**N** (0.99) /Y (0.004) /I (0.003)	0.59/0.08
258	HA	158	**N** (0.94) /K (0.05) /H (0.006)	**N** (0.98) /K (0.008) /I (0.002)	0.35/0.1
18	NA	354	**F** (0.85)/S(0.13)	**F** (0.98)/V(0.004)	0.61/0.09
48	NA	98	**A** (0.94)/V(0.05)/E(0.002)	**A**(0.95)/V(0.04)/G(0.002)	0.29/0.25
49	NA	221	**D** (0.86)/E(0.12)/A(0.005)	**D** (0.98)/A(0.005)/E(0.002)	0.6/0.08
52	NA	197	**D**(0.86)/G(0.12)/E(0.003)	**D** (0.98)/E(0.005)/Y(0.002	0.6/0.07
60	NA	41	**K** (0.96)/E (0.03)	**E** (0.99)/D (0.004)	0.24/0.06
60	NA	335	**S** (0.906) /R (0.09) /T (0.001)	**S** (0.99) /R (0.003) /T (0.002)	0.45/0.06
85	NA	349	**M** (0.94)/V (0.05)	**V** (0.99)/G (0.003)	0.32/0.06
89	NA	44	**S** (0.97) /P (0.02) /T (0.004)	**S** (0.91) /P (0.09) /T (0.002)	0.18/0.45
15	PB2	732	**V** (0.94) /G (0.05) /L (0.004)	**V** (0.96) /G (0.04) /L (0.004)	0.33/0.26
15	PB2	733	**L** (0.94) /W (0.021) /V (0.015)	**L** (0.97) /W (0.012) /V (0.009)	0.29/0.19
15	PB2	738	**K** (0.95) /N (0.04) /T (0.006)	**K** (0.96) /N (0.03) /T (0.004)	0.29/0.22
19	PB2	375	**R** (0.93) /K (0.067) /T (0.001)	**R** (0.99) /T (0.001)	0.37/0.03
19	PB2	728	**Q** (0.94) /K (0.04) /H (0.02)	**Q** (0.97) /H (0.02) /L (0.004)	0.36/0.18
2	PB2	732	**V** (0.95) /G (0.04) /L (0.003)	**V** (0.95) /G (0.04) /L (0.004)	0.27/0.3
35	PB2	154	**L** (0.79) /I (0.2) /F (0.001)	**L** (0.99) /I (0.002) /P (0.001)	0.74/0.04
35	PB2	28	**M** (0.95) /V (0.05) /I (0.003)	**M** (0.99) /I (0.004) /K (0.003)	0.31/0.07
35	PB2	99	**W** (0.84) /C (0.16) /G (0.001)	**W** (0.99) /G (0.002) /C (0.002)	0.65/0.05
36	PB2	451	**V** (0.93) /I (0.07) /G (0.002)	**V** (0.99 /I (0.003) /G (0.001)	0.37/0.05
37	PB2	732	**V** (0.95) /G (0.05) /L (0.003)	**V** (0.95) /G (0.04) /L (0.004)	0.31/0.29
42	PB2	336	**S** (0.96) /A (0.04) /T (0.002)	**S** (0.94) /A (0.05) /P (0.008)	0.24/0.34
42	PB2	92	**S** (0.92) /P (0.08)	**S** (0.64) /P (0.36) /T (0.001)	0.42/0.96
48	PB2	732	**V** (0.95) /G (0.04) /L (0.003)	**V** (0.95) /G (0.04) /L (0.004)	0.28/0.30
52	PB2	261	**I** (0.94) /T (0.05) /N (0.001)	**I** (0.99) /N (0.002) /L (0.001)	0.32/0.03
52	PB2	471	**T** (0.81) /I (0.19) /S (0.002)	**T** (0.99) /S (0.002) /R (0.002)	0.71/0.04
52	PB2	691	**L** (0.94) /S (0.06) /F (0.001)	**L** (0.99) /F (0.001) /V (0.001)	0.34/0.03
52	PB2	732	**V** (0.96) /G (0.04) /L (0.003)	**V** (0.95) /G (0.05) /A (0.003)	0.25/0.31
55	PB2	227	**I** (0.99) /V (0.002) /L (0.001)	**I** (0.95) /M (0.05) /L (0.001)	0.03/0.28
55	PB2	732	**V** (0.95) /G (0.04) /L (0.003)	**V** (0.95) /G (0.05) /L (0.003)	0.27/0.31
60	PB2	733	**L** (0.92) /V (0.04) /W (0.013)	**L** (0.97) /W (0.013) /V (0.01)	0.37/0.20

80	PB2	315	**M** (0.88) /T (0.119) /I (0.002)	**M** (0.99) /L (0.003) /I (0.002)	0.55/0.05
85	PB2	369	**R** (0.69) /K (0.3) /S (0.003)	**R** (0.99) /S (0.004) /G (0.002)	0.91/0.06
85	PB2	442	**A** (0.89) /V (0.104) /S (0.002)	**A** (0.99) /S (0.004) /E (0.001)	0.51/0.04
85	PB2	58	**T** (0.95) /N (0.05) /S (0.001)	**T** (0.99) /S (0.001) /A (0.001)	0.3/0.02
85	PB2	631	**I** (0.94) /M (0.06) /K (0.002)	**M** (0.99) /K (0.002) /I (0.001)	0.36/0.04
89	PB2	336	**S** (0.95) /A (0.04) /P(0.002)	**S** (0.95) /A (0.03) /P (0.001)	0.29/0.2
89	PB2	732	**V** (0.95) /G (0.04) /L (0.004)	**V** (0.96) /G (0.03) /L (0.002)	0.3/0.22
89	PB2	733	**L** (0.95) /V (0.02) /W (0.03)	**L** (0.97) /V (0.01) /W (0.01)	0.27/0.18
92	PB2	732	**V** (0.95) /G (0.041) /L (0.003)	**V** (0.95) /G (0.043) /L (0.003)	0.28/0.3
101	PB2	47	**M** (0.80) /I (0.19) /V (0.001)	**M** (0.99) /I (0.01)	0.73/0.09
102	PB2	588	**T** (0.99) /A (0.004) /P (0.002)	**T** (0.94) /N (0.06) /A (0.003)	0.06/0.34
12	PB1	375	**S** (0.99) /R (0.004) /C (0.002)	**S** (0.95) /G (0.04) /R (0.004)	0.06/0.30
13	PB1	540	**G** (0.91) /R (0.09) /S (0.001)	**G** (0.99) /R (0.002)	0.44/0.04
15	PB1	215	**R** (0.78) /K (0.21) /S (0.006)	**R** (0.73) /K (0.26) /S (0.002)	0.8/0.86
2	PB1	654	**S** (0.93) /G (0.06) /R (0.003)	**S** (0.99) /C (0.002) /R (0.002)	0.36/0.05
35	PB1	51	**E** (0.78) /K (0.212)	**E** (0.99) /D (0.001) /A (0.001)	0.76/0.03
35	PB1	99	**H** (0.92) /N (0.07) /P (0.01)	**H** (0.98) /P (0.01) /L (0.002)	0.46/0.10
36	PB1	312	**N** (0.92) /T (0.08) /K (0.002)	**N** (0.99) /Y (0.001) /K (0.001)	0.41/0.04
40	PB1	707	**R** (0.98) /I (0.011)	**R** (0.87) /I (0.125) /S (0.006)	0.15/0.60
42	PB1	697	**E** (0.99) /D (0.002) /K (0.001)	**E** (0.91) /K (0.087) /D (0.001)	0.04/0.45
42	PB1	701	**P** (0.82) /L (0.17) /R (0.002)	**P** (0.68) /L (0.32) /T (0.001)	0.68/0.91
42	PB1	83	**A** (0.99) /T (0.006)	**A** (0.94) /T (0.057) /S (0.004)	0.08/0.36
48	PB1	111	**M** (0.90) /I (0.098) /K (0.002)	**M** (0.99) /K (0.002) /I (0.002)	0.49/0.04
51	PB1	406	**G** (0.91) /E (0.08) /V (0.003)	**G** (0.992) /V (0.002)	0.44/0.05
55	PB1	111	**M** (0.90) /I (0.10) /L (0.003)	**M** (0.96) /I (0.033) /L (0.002)	0.48/0.24
55	PB1	281	**K** (0.93) /Q (0.07) /N (0.004)	**K** (0.98) /N (0.006) /I (0.004)	0.39/0.10
58	PB1	181	**I** (0.65) /R (0.35) /S (0.006)	**I** (0.99) /K (0.004) /L (0.003)	0.98/0.07
58	PB1	251	**F** (0.92) /S (0.08)	**F** (0.98) /V (0.005) /L (0.004)	0.41/0.09
58	PB1	375	**S** (1)	**S** (0.93) /G (0.06) /R (0.004)	0.00/0.37
58	PB1	670	**G** (0.53) /R (0.5) /K (0.01)	**R** (0.99) /S (0.003) /G (0.003)	1.04/0.06
58	PB1	740	**F** (0.95) /C (0.029) /V (0.013)	**F** (0.98) /C (0.01) /V (0.006)	0.30/0.14
60	PB1	527	**V** (0.87) /I (0.127) /L (0.002)	**V** (0.99) /L (0.002) /E (0.002)	0.58/0.04
76	PB1	151	**R** (0.99) /S (0.003) /I (0.001)	**R** (0.94) /K (0.057) /S (0.002)	0.04/0.34
76	PB1	532	**N** (0.94) /K (0.051) /H (0.003)	**N** (0.99) /H (0.003) /K (0.002)	0.33/0.05
77	PB1	678	**S** (0.99) /R (0.006) /T (0.002)	**S** (0.91) /T (0.082) /R (0.004)	0.08/0.46
78	PB1	707	**R** (0.99) /S (0.007)	**R** (0.87) /T (0.118) /S (0.007)	0.08/0.59
80	PB1	152	**S** (0.92) /P (0.07)	**S** (0.99) /A (0.001) /T (0.001)	0.40/0.03
83	PB1	30	**Y** (0.99) /N (0.003) /S (0.001)	**Y** (0.68) /H (0.309) /N (0.002)	0.04/0.92
85	PB1	229	**K** (0.81) /N (0.18) /E (0.002)	**K** (0.99) /N (0.003)	0.71/0.06
85	PB1	440	**G** (0.92) /E (0.07) /W (0.005)	**G** (0.99) /W (0.004) /R (0.003)	0.43/0.07
85	PB1	709	**I** (0.93) /V (0.04) /A (0.02)	**I** (0.99) /F (0.003) /N (0.002)	0.41/0.06
101	PB1	148	**E** (0.93) /V (0.07) /D (0.002)	**E** (0.99) /V (0.006)	0.39/0.08
102	PB1	302	**I** (0.99) /F (0.002) /M (0.001)	**V** (0.53) /I (0.46) /G (0.001)	0.03/1.01
102	PB1	408	**M** (0.99) /L (0.003) /I (0.002)	**M** (0.95) /I (0.04) /L (0.003)	0.06/0.29
102	PB1	492	**F** (0.99) /I (0.002) /L (0.002)	**L** (0.51) /F (0.49) /I (0.001)	0.05/1.01
102	PB1	581	**D** (0.93) /G (0.07) /E (0.003)	**D** (0.99) /E (0.003) /Y (0.001)	0.39/0.05
8	PA	400	**L** (0.95) /K (0.03) /I (0.02)	**L** (0.95) /P (0.03) /K (0.02)	0.33/0.33
12	PA	400	**L** (0.97) /P (0.02) /K (0.009)	**L** (0.95) /P (0.03) /K (0.02)	0.21/0.32
13	PA	609	**K** (0.94) /N (0.06) /I (0.002)	**K** (0.99) /N (0.002) /I (0.001)	0.35/0.04
15	PA	400	**L** (0.96) /P (0.022) /K (0.006)	**L** (0.95) /P (0.04) /K (0.01)	0.22/0.32
18	PA	400	**L** (1.00)	**L** (0.90) /K (0.04) /P (0.03)	0.00/0.48
18	PA	404	**S** (0.75) /A (0.25)	**S** (0.93) /A (0.06) /T (0.003)	0.81/0.37
18	PA	573	**V** (0.96) /I (0.04) /D (0.001)	**V** (0.97) /I (0.02) /L (0.001)	0.26/0.18
18	PA	643	**K** (0.91) /R (0.091) /N (0.002)	**K** (0.99) /N (0.003)	0.46/0.05
19	PA	400	**L** (0.93) /F (0.04) /K (0.02)	**L** (0.93) /K (0.06) /P (0.006)	0.41/0.38
19	PA	404	**S** (0.87) /A (0.13)	**S** (0.83) /A (0.17) /T (0.005)	0.56/0.69
2	PA	400	**L** (0.73) /P (0.126) /K (0.13)	**L** (0.78) /K (0.13) /P (0.091)	1.08/0.97
2	PA	404	**S** (0.81) /A (0.18) /T (0.009)	**S** (0.84) /A (0.16) /P (0.006)	0.75/0.68
35	PA	400	**L** (0.62) /K (0.23) /I (0.08)	**L** (0.77) /P (0.15) /K (0.06)	1.20/0.96

35	PA	402	**S** (0.92) /A (0.04) /T (0.04)	**S** (0.96) /T (0.02) /A (0.02)	0.47/0.31
35	PA	404	**S** (0.94) /A (0.07)	**S** (0.80) /A (0.19) /T (0.01)	0.35/0.81
35	PA	442	**R** (0.92) /G (0.08) /I (0.002)	**R** (0.99) /S (0.002) /I (0.001)	0.42/0.04
36	PA	400	**L** (0.75) /K (0.25)	**L** (0.86) /K (0.05) /P (0.04)	0.81/0.61
36	PA	404	**S** (0.8) /A (0.1) /T (0.1)	**S** (0.78) /A (0.205) /T (0.016)	0.92/0.85
37	PA	400	**L** (0.73) /K (0.18) /P (0.09)	**L** (0.81) /P (0.14) /I (0.02)	1.10/0.76
37	PA	402	**S** (0.95) /L (0.018) /T (0.018)	**S** (0.96) /T (0.02) /L (0.01)	0.28/0.24
37	PA	404	**S** (0.81) /A (0.18) /T (0.012)	**S** (0.82) /A (0.19)	0.77/0.69
40	PA	400	**L** (0.8) /K (0.07) /P (0.07)	**L** (0.83) /P (0.08) /K (0.08)	0.78/0.82
40	PA	404	**S** (0.88) /A (0.13)	**S** (0.90) /A (0.11)	0.54/0.49
42	PA	400	**L** (0.95) /P (0.03) /K (0.008)	**L** (1)	0.28/0.00
42	PA	404	**S** (0.98) /A (0.017) /T (0.003)	**S** (0.94) /A (0.047) /T (0.012)	0.16/0.36
51	PA	400	**L** (0.88) /K (0.07) /F (0.02)	**L** (0.93) /K (0.04) /F (0.013)	0.54/0.36
51	PA	404	**S** (0.89) /A (0.11) /T (0.004)	**S** (0.86) /A (0.13) /T (0.008)	0.53/0.63
52	PA	142	**N** (0.88) /D (0.11) /K (0.003)	**N** (0.98) /H (0.005) /K (0.005)	0.53/0.10
52	PA	383	**N** (0.99) /Y (0.004)	**N** (0.95) /D (0.04)	0.04/0.26
52	PA	396	**E** (0.96) /D (0.04)	**E** (1)	0.26/0.00
52	PA	400	**L** (0.77) /K (0.09) /P (0.09)	**L** (0.86) /K (0.14)	0.92/0.59
52	PA	404	**S** (0.84) /A (0.16)	**S** (0.65) /A (0.35)	0.63/0.93
58	PA	332	**S** (0.72) /P (0.28) /F (0.001)	**S** (0.99) /T (0.001)	0.87/0.01
58	PA	400	**L** (0.97) /K (0.032)	**L** (0.95) /P (0.03) /K (0.01)	0.21/0.27
58	PA	531	**R** (0.87) /G (0.12) /S (0.002)	**R** (0.99) /S (0.003) /G (0.001)	0.56/0.04
60	PA	400	**L** (0.92) /K (0.08)	**L** (0.95) /K (0.02) /P (0.02)	0.39/0.28
60	PA	404	**S** (0.84) /A (0.12) /T (0.04)	**S** (0.85) /A (0.14) /T (0.01)	0.76/0.67
60	PA	461	**K** (0.92) /N (0.071) /I (0.003)	**K** (0.99) /N (0.002) /I (0.001)	0.40/0.03
62	PA	332	**S** (0.99) /P (0.001)	**S** (0.51) /T (0.49) /P (0.001)	0.01/1.01
62	PA	614	**N** (0.93) /K (0.07) /I (0.002)	**N** (0.99) /K (0.002) /D (0.002)	0.37/0.04
80	PA	325	**P** (0.84) /Q (0.16) /T (0.001)	**P** (0.99) /T (0.001)	0.65/0.01
80	PA	674	**D** (0.95) /G (0.05) /E (0.002)	**D** (0.99) /G (0.002) /A (0.002)	0.3/0.04
80	PA	704	**A** (0.93) /V (0.07) /E (0.001)	**A** (0.99) /E (0.001) /G (0.001)	0.38/0.02
83	PA	400	**L** (0.86) /K (0.09) /P (0.03)	**L** (0.89) /K (0.06) /P (0.03)	0.64/0.53
83	PA	404	**S** (0.86) /A (0.13) /T (0.007)	**S** (0.88) /A (0.12) /T (0.005)	0.62/0.56
85	PA	385	**R** (0.95) /K (0.05)	**R** (0.98) /K (0.02) /I (0.001)	0.28/0.16
85	PA	396	**E** (0.86) /D (0.14)	**E** (0.98) /D (0.019)	0.59/0.14
85	PA	400	**L** (1)	**L** (0.91) /K (0.06) /P (0.023)	0.00/0.49
85	PA	402	**S** (0.91) /A (0.091)	**S** (0.96) /A (0.022) /T (0.017)	0.44/0.28
85	PA	404	**S** (0.93) /A (0.071)	**S** (0.93) /A (0.068)	0.37/0.36
85	PA	573	**V** (0.95) /I (0.04) /T (0.002)	**V** (0.97) /I (0.027) /L (0.001)	0.28/0.19
85	PA	614	**N** (0.81) /D (0.19) /Y (0.003)	**N** (0.99) /K (0.002) /Y (0.001)	0.72/0.04
85	PA	400	**L** (0.96) /P (0.021) /K (0.011)	**L** (0.96) /P (0.021) /K (0.014)	0.25/0.26
92	PA	400	**L** (0.85) /P (0.084) /K (0.07)	**L** (0.89) /P (0.077) /I (0.04)	0.76/0.62
92	PA	402	**S** (0.93) /T (0.04) /A (0.021)	**S** (0.97) /T (0.017) /P (0.017)	0.4/0.24
92	PA	404	**S** (0.79) /A (0.20) /T (0.011)	**S** (0.83) /A (0.15) /T (0.02)	0.81/0.76
101	PA	385	**R** (0.94) /K (0.06)	**R** (0.99) /K (0.013)	0.31/0.1
101	PA	396	**E** (1)	**E** (0.94) /D (0.06)	0.00/0.32
101	PA	400	**L** (0.75) /P (0.17) /K (0.08)	**L** (0.81) /K (0.16) /P (0.03)	1.04/0.81
15	NP	351	**K** (0.94) /R (0.05) /S (0.009)	**K** (0.99) /R (0.003) /S (0.003)	0.35/0.06
37	NP	370	**N** (0.92) /S (0.07) /I (0.001)	**N** (0.97) /S (0.033) /K (0.001)	0.39/0.22
48	NP	208	**R** (0.99) /S (0.002)	**R** (0.93) /K (0.062) /G (0.001)	0.04/0.35
52	NP	431	**G**(0.62)/E(0.37)/(0.004)	**G**(1)/R(0.002)/(0.001)	0.99/0.03
58	NP	103	**K**(0.92)/E(0.072)/N(0.002)	**K**(0.99)/N(0.003)/E(0.001)	0.40/0.04
58	NP	170	**S**(0.94)/P(0.052)/A(0.006)	**S**(0.99)/A(0.007)/P(0.001)	0.35/0.07
62	NP	231	**Q**(0.93)/R(0.067)/H(0.002)	**Q**(0.99)/H(0.002)/L(0.001)	0.38/0.04
62	NP	404	**G**(0.89)/D(0.105)/A(0.002)	**G**(1)/A(0.001)/R(0.001)	0.51/0.02
77	NP	105	**M**(0.99)/I(0.01)/R(0.002)	**M**(0.84)/I(0.152)/R(0.001)	0.10/0.63
78	NP	239	**V**(0.99)/M(0.005)/G(0.004)	**V**(0.88)/M(0.12)/G(0.003)	0.08/0.56
78	NP	319	**N**(0.997)/K(0.002)/I(0.001)	**N**(0.8)/K(0.196)/I(0.001)	0.04/0.73
79	NP	324	**H**(0.89)/Q(0.107)/L(0.001)	**H**(1)/L(0.001)/P(0.001)	0.50/0.02
79	NP	325	**K**(0.89)/E(0.107)/R(0.001)	**K**(0.99)/R(0.001)/N(0.001)	0.51/0.03

85	NP	418	**L**(0.93)/P(0.066)/H(0.001)	**L**(1)/P(0.001)/H(0.001)	0.36/0.03
102	NP	242	**V**(1)/G(0.001)/D(0.001)	**V**(0.73)/I(0.26)/F(0.001)	0.03/0.84
35	MP	220	**G** (0.95) /R (0.05) /V (0.002)	**G** (0.99) /V (0.002) /R (0.002)	0.31/0.04
35	MP	68	**V** (0.88) /M (0.11) /L (0.002)	**V** (0.99) /E (0.003) /L (0.002)	0.52/0.05
36	MP	140	**T** (0.93) /I (0.06) /S (0.003)	**T** (0.99) /S (0.003) /P (0.002)	0.36/0.05
51	MP	19	**C** (0.62) /Y (0.37) /S (0.002)	**C** (0.65) /Y (0.34) /W (0.001)	0.97/0.95
51	MP	55	**P** (0.94) /L (0.05) /A (0.002)	**P** (0.904) /L (0.09) /R (0.002)	0.33/0.46
13	NS2	102	**Q** (0.94) /L (0.06) /E (0.001)	**Q** (0.99) /E (0.001) /H (0.001)	0.34/0.03
35	NS2	77	**R** (0.92) /G (0.08) /I (0.001)	**R** (0.99) /G (0.001) /S (0.001)	0.41/0.02
36	NS2	102	**Q** (0.91) /H(0.09)	**Q** (0.99) /E (0.001) /H (0.001)	0.46/0.03
51	NS2	93	**A** (0.93) /E (0.06) /S (0.001)	**A** (0.99) /S (0.001) /G (0.001)	0.36/0.02
55	NS2	13	**G** (0.94) /E (0.052) /V (0.002)	**G** (0.99) /R (0.003) /V (0.002)	0.32/0.06
58	NS1	180	**V** (0.92) /A (0.07) /G (0.007)	**V** (0.98) /G (0.01) /A (0.002)	0.43/0.11
58	NS2	14	**S** (0.92) /P (0.07) /A (0.008)	**S** (0.98) /A (0.01) /P (0.002)	0.43/0.11
58	NS2	53	**N** (0.92) /S (0.07) /T (0.002)	**N** (0.98) /T (0.003) /I (0.002)	0.39/0.06
60	NS1	150	**F** (0.91) /L (0.09) /I (0.002)	**F** (0.99) /L (0.004) /I (0.002)	0.46/0.06
79	NS1	129	**M** (0.95) /T (0.04) /I (0.004)	**M** (0.99) /I (0.004) /K (0.001)	0.29/0.06
85	NS1	181	**L** (0.94) /F (0.05) /I (0.002)	**L** (0.99) /I (0.002) /P (0.002)	0.32/0.04
101	NS1	81	**M** (0.99) /I (0.005) /L (0.002)	**M** (0.95) /K (0.05) /I (0.005)	0.06/0.32

FIGURE S1. Time-scaled Bayesian phylogenetic trees of the six internal gene segments PB2 (a), PB1 (b), PA (c), NP (d), M (e), and NS (f) from H3N2 influenza viruses collected during the 2017–2018 influenza season. Clinical samples are shown in red, and their corresponding hCK-derived isolates in blue; representative circulating H3N2 strains retrieved from the GISAID EpiFlu database (collected up to 2018) are shown in black for clade context. Phylogenetic trees were inferred using BEAST v1.10.5 under an HKY substitution model with a unrelated relaxed clock and a coalescent exponential growth prior. Posterior probabilities ≥0.70 are indicated at key nodes.

FIGURE S2. Comparison of intrahost genetic diversity between clinical H3N2 specimens and their matched hCK-derived isolates across internal gene segments. Shannon entropy values, derived from deep sequencing data, are plotted for each amino acid position of PB2 (a), PB1 (b), PA (c), NP (d), M (e), and NS (f). Red circles represent clinical samples, while blue triangles denote corresponding isolates. Higher entropy values reflect greater amino acid diversity within a sample. Across all segments, clinical specimens generally exhibited greater variability than their matched isolates, indicating that virus culture imposes purifying selection and reduces within-sample diversity. Entropy values were calculated from iSNVs identified using DiversiTools (https://github.com/josephhughes/DiversiTools).

FIG S1a PB2

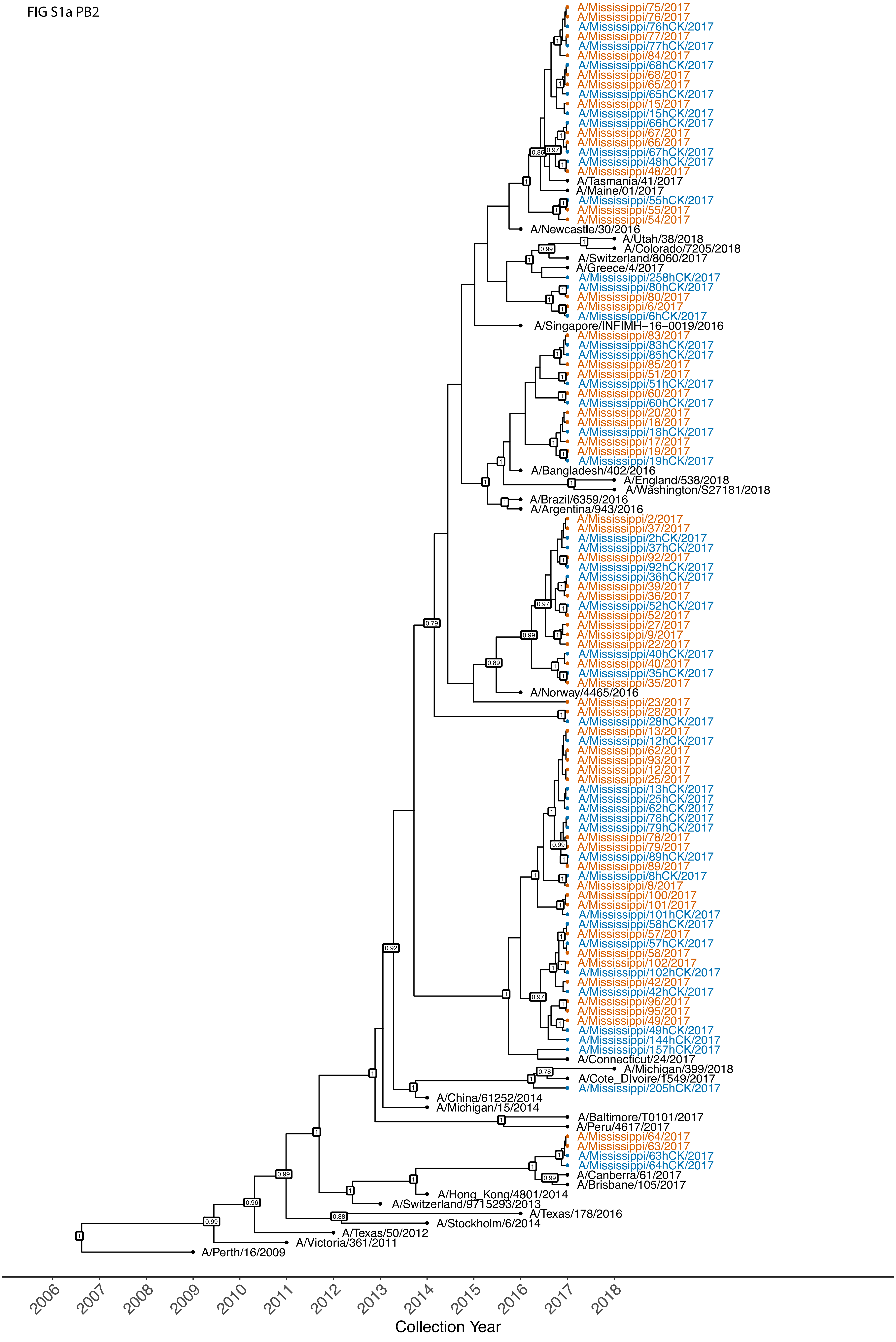
A/Mississippi/75/2017
A/Mississippi/76/2017
A/Mississippi/76hCK/2017
A/Mississippi/77/2017
A/Mississippi/77hCK/2017
A/Mississippi/84/2017
A/Mississippi/68hCK/2017
A/Mississippi/68/2017
A/Mississippi/65/2017
A/Mississippi/65hCK/2017
A/Mississippi/15/2017
A/Mississippi/15hCK/2017
A/Mississippi/66hCK/2017
A/Mississippi/67/2017
A/Mississippi/66/2017
A/Mississippi/67hCK/2017
A/Mississippi/48hCK/2017
A/Mississippi/48/2017
A/Tasmania/41/2017
A/Maine/01/2017
A/Mississippi/55hCK/2017
A/Mississippi/55/2017
A/Mississippi/54/2017
A/Newcastle/30/2016
A/Utah/38/2018
A/Colorado/7205/2018
A/Switzerland/8060/2017
A/Greece/4/2017
A/Mississippi/258hCK/2017
A/Mississippi/80hCK/2017
A/Mississippi/80/2017
A/Mississippi/6/2017
A/Mississippi/6hCK/2017
A/Singapore/INFIMH-16-0019/2016
A/Mississippi/83/2017
A/Mississippi/83hCK/2017
A/Mississippi/85hCK/2017
A/Mississippi/85/2017
A/Mississippi/51/2017
A/Mississippi/51hCK/2017
A/Mississippi/60/2017
A/Mississippi/60hCK/2017
A/Mississippi/20/2017
A/Mississippi/18/2017
A/Mississippi/18hCK/2017
A/Mississippi/17/2017
A/Mississippi/19/2017
A/Mississippi/19hCK/2017
A/Bangladesh/402/2016
A/England/538/2018
A/Washington/S27181/2018
A/Brazil/6359/2016
A/Argentina/943/2016
A/Mississippi/2/2017
A/Mississippi/37/2017
A/Mississippi/2hCK/2017
A/Mississippi/37hCK/2017
A/Mississippi/92/2017
A/Mississippi/92hCK/2017
A/Mississippi/36hCK/2017
A/Mississippi/39/2017
A/Mississippi/36/2017
A/Mississippi/52hCK/2017
A/Mississippi/52/2017
A/Mississippi/27/2017
A/Mississippi/9/2017
A/Mississippi/22/2017
A/Mississippi/40hCK/2017
A/Mississippi/40/2017
A/Mississippi/35hCK/2017
A/Mississippi/35/2017
A/Norway/4465/2016
A/Mississippi/23/2017
A/Mississippi/28/2017
A/Mississippi/28hCK/2017
A/Mississippi/13/2017
A/Mississippi/12hCK/2017
A/Mississippi/62/2017
A/Mississippi/93/2017
A/Mississippi/12/2017
A/Mississippi/25/2017
A/Mississippi/13hCK/2017
A/Mississippi/25hCK/2017
A/Mississippi/62hCK/2017
A/Mississippi/78hCK/2017
A/Mississippi/79hCK/2017
A/Mississippi/78/2017
A/Mississippi/79/2017
A/Mississippi/89hCK/2017
A/Mississippi/89/2017
A/Mississippi/8hCK/2017
A/Mississippi/8/2017
A/Mississippi/100/2017
A/Mississippi/101/2017
A/Mississippi/101hCK/2017
A/Mississippi/58hCK/2017
A/Mississippi/57/2017
A/Mississippi/57hCK/2017
A/Mississippi/58/2017
A/Mississippi/102/2017
A/Mississippi/102hCK/2017
A/Mississippi/42/2017
A/Mississippi/42hCK/2017
A/Mississippi/96/2017
A/Mississippi/95/2017
A/Mississippi/49/2017
A/Mississippi/49hCK/2017
A/Mississippi/144hCK/2017
A/Mississippi/157hCK/2017
A/Connecticut/24/2017
A/Michigan/399/2018
A/Cote_DIvoire/1549/2017
A/Mississippi/205hCK/2017
A/China/61252/2014
A/Michigan/15/2014
A/Baltimore/T0101/2017
A/Peru/4617/2017
A/Mississippi/64/2017
A/Mississippi/63/2017
A/Mississippi/63hCK/2017
A/Mississippi/64hCK/2017
A/Canberra/61/2017
A/Brisbane/105/2017
A/Hong_Kong/4801/2014
A/Switzerland/9715293/2013
A/Texas/178/2016
A/Stockholm/6/2014
A/Texas/50/2012
A/Victoria/361/2011
A/Perth/16/2009
2006
2007
2008
2009
2010
2011
2012
2013
2014
2015
2016
2017
2018
Collection Year

FIG. S1b PB1

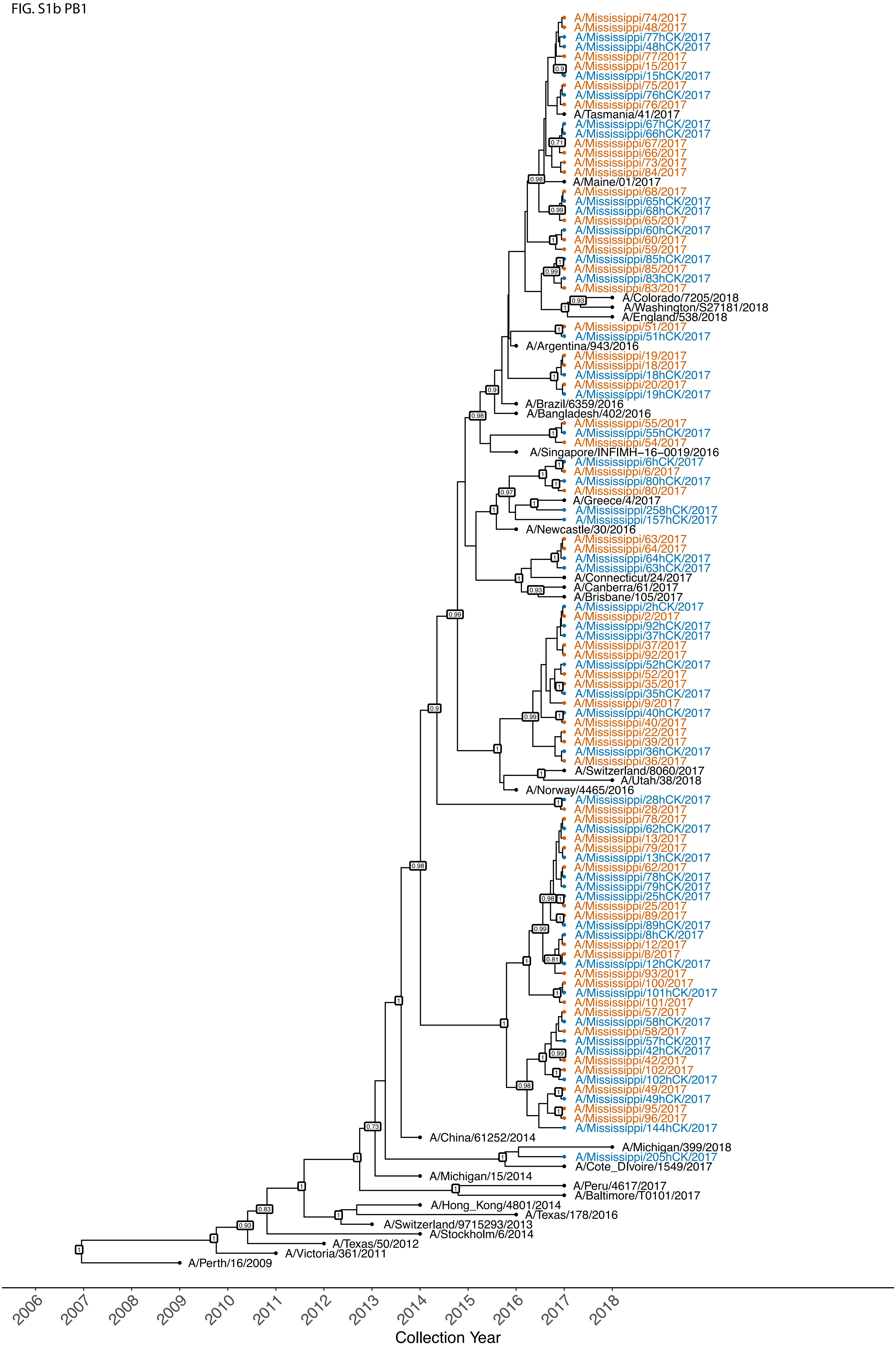
A/Mississippi/74/2017
A/Mississippi/48/2017
A/Mississippi/77hCK/2017
A/Mississippi/48hCK/2017
A/Mississippi/77/2017
A/Mississippi/15/2017
A/Mississippi/15hCK/2017
A/Mississippi/75/2017
A/Mississippi/76hCK/2017
A/Mississippi/76/2017
A/Tasmania/41/2017
A/Mississippi/67hCK/2017
A/Mississippi/66hCK/2017
A/Mississippi/67/2017
A/Mississippi/66/2017
A/Mississippi/73/2017
A/Mississippi/84/2017
A/Maine/01/2017
A/Mississippi/68/2017
A/Mississippi/65hCK/2017
A/Mississippi/68hCK/2017
A/Mississippi/65/2017
A/Mississippi/60hCK/2017
A/Mississippi/60/2017
A/Mississippi/59/2017
A/Mississippi/85hCK/2017
A/Mississippi/85/2017
A/Mississippi/83hCK/2017
A/Mississippi/83/2017
A/Colorado/7205/2018
A/Washington/S27181/2018
A/England/538/2018
A/Mississippi/51/2017
A/Mississippi/51hCK/2017
A/Argentina/943/2016
A/Mississippi/19/2017
A/Mississippi/18/2017
A/Mississippi/18hCK/2017
A/Mississippi/20/2017
A/Mississippi/19hCK/2017
A/Brazil/6359/2016
A/Bangladesh/402/2016
A/Mississippi/55/2017
A/Mississippi/55hCK/2017
A/Mississippi/54/2017
A/Singapore/INFIMH−16−0019/2016
A/Mississippi/6hCK/2017
A/Mississippi/6/2017
A/Mississippi/80hCK/2017
A/Mississippi/80/2017
A/Greece/4/2017
A/Mississippi/258hCK/2017
A/Mississippi/157hCK/2017
A/Newcastle/30/2016
A/Mississippi/63/2017
A/Mississippi/64/2017
A/Mississippi/64hCK/2017
A/Mississippi/63hCK/2017
A/Connecticut/24/2017
A/Canberra/61/2017
A/Brisbane/105/2017
A/Mississippi/2hCK/2017
A/Mississippi/2/2017
A/Mississippi/92hCK/2017
A/Mississippi/37hCK/2017
A/Mississippi/37/2017
A/Mississippi/92/2017
A/Mississippi/52hCK/2017
A/Mississippi/52/2017
A/Mississippi/35/2017
A/Mississippi/35hCK/2017
A/Mississippi/9/2017
A/Mississippi/40hCK/2017
A/Mississippi/40/2017
A/Mississippi/22/2017
A/Mississippi/39/2017
A/Mississippi/36hCK/2017
A/Mississippi/36/2017
A/Switzerland/8060/2017
A/Utah/38/2018
A/Norway/4465/2016
A/Mississippi/28hCK/2017
A/Mississippi/28/2017
A/Mississippi/78/2017
A/Mississippi/62hCK/2017
A/Mississippi/13/2017
A/Mississippi/79/2017
A/Mississippi/13hCK/2017
A/Mississippi/62/2017
A/Mississippi/78hCK/2017
A/Mississippi/79hCK/2017
A/Mississippi/25hCK/2017
A/Mississippi/25/2017
A/Mississippi/89/2017
A/Mississippi/89hCK/2017
A/Mississippi/8hCK/2017
A/Mississippi/12/2017
A/Mississippi/8/2017
A/Mississippi/12hCK/2017
A/Mississippi/93/2017
A/Mississippi/100/2017
A/Mississippi/101hCK/2017
A/Mississippi/101/2017
A/Mississippi/57/2017
A/Mississippi/58hCK/2017
A/Mississippi/58/2017
A/Mississippi/57hCK/2017
A/Mississippi/42hCK/2017
A/Mississippi/42/2017
A/Mississippi/102/2017
A/Mississippi/102hCK/2017
A/Mississippi/49/2017
A/Mississippi/49hCK/2017
A/Mississippi/95/2017
A/Mississippi/96/2017
A/Mississippi/144hCK/2017
A/China/61252/2014
A/Michigan/399/2018
A/Mississippi/205hCK/2017
A/Cote_DIvoire/1549/2017
A/Michigan/15/2014
A/Peru/4617/2017
A/Baltimore/T0101/2017
A/Hong_Kong/4801/2014
A/Texas/178/2016
A/Switzerland/9715293/2013
A/Stockholm/6/2014
A/Texas/50/2012
A/Victoria/361/2011
A/Perth/16/2009
2006
2007
2008
2009
2010
2011
2012
2013
2014
2015
2016
2017
2018
Collection Year

FIG. S1c PA

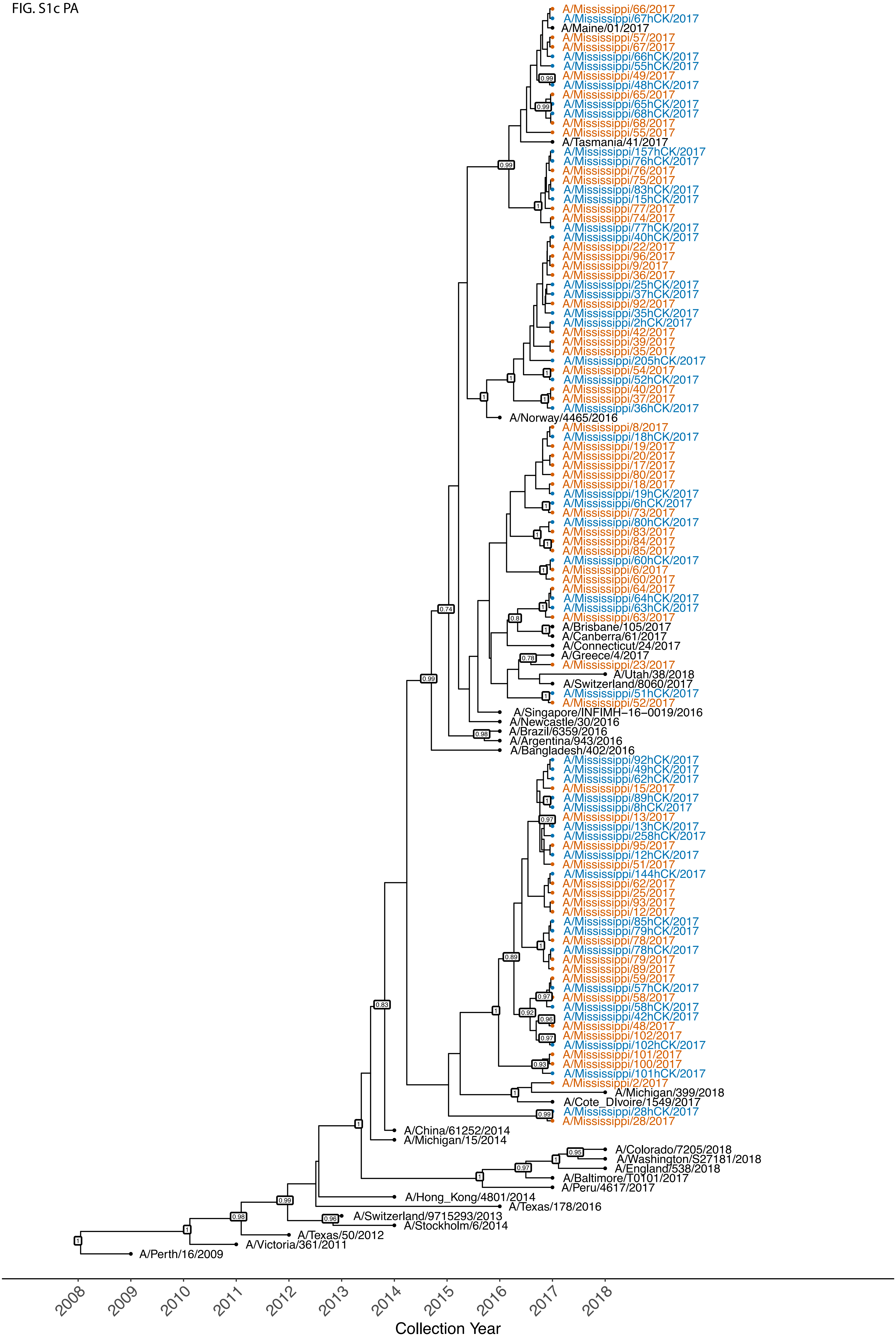
A/Mississippi/66/2017
A/Mississippi/67hCK/2017
A/Maine/01/2017
A/Mississippi/57/2017
A/Mississippi/67/2017
A/Mississippi/66hCK/2017
A/Mississippi/55hCK/2017
A/Mississippi/49/2017
A/Mississippi/48hCK/2017
A/Mississippi/65/2017
A/Mississippi/65hCK/2017
A/Mississippi/68hCK/2017
A/Mississippi/68/2017
A/Mississippi/55/2017
A/Tasmania/41/2017
A/Mississippi/157hCK/2017
A/Mississippi/76hCK/2017
A/Mississippi/76/2017
A/Mississippi/75/2017
A/Mississippi/83hCK/2017
A/Mississippi/15hCK/2017
A/Mississippi/77/2017
A/Mississippi/74/2017
A/Mississippi/77hCK/2017
A/Mississippi/40hCK/2017
A/Mississippi/22/2017
A/Mississippi/96/2017
A/Mississippi/9/2017
A/Mississippi/36/2017
A/Mississippi/25hCK/2017
A/Mississippi/37hCK/2017
A/Mississippi/92/2017
A/Mississippi/35hCK/2017
A/Mississippi/2hCK/2017
A/Mississippi/42/2017
A/Mississippi/39/2017
A/Mississippi/35/2017
A/Mississippi/205hCK/2017
A/Mississippi/54/2017
A/Mississippi/52hCK/2017
A/Mississippi/40/2017
A/Mississippi/37/2017
A/Mississippi/36hCK/2017
A/Norway/4465/2016
A/Mississippi/8/2017
A/Mississippi/18hCK/2017
A/Mississippi/19/2017
A/Mississippi/20/2017
A/Mississippi/17/2017
A/Mississippi/80/2017
A/Mississippi/18/2017
A/Mississippi/19hCK/2017
A/Mississippi/6hCK/2017
A/Mississippi/73/2017
A/Mississippi/80hCK/2017
A/Mississippi/83/2017
A/Mississippi/84/2017
A/Mississippi/85/2017
A/Mississippi/60hCK/2017
A/Mississippi/6/2017
A/Mississippi/60/2017
A/Mississippi/64/2017
A/Mississippi/64hCK/2017
A/Mississippi/63hCK/2017
A/Mississippi/63/2017
A/Brisbane/105/2017
A/Canberra/61/2017
A/Connecticut/24/2017
A/Greece/4/2017
A/Mississippi/23/2017
A/Utah/38/2018
A/Switzerland/8060/2017
A/Mississippi/51hCK/2017
A/Mississippi/52/2017
A/Singapore/INFIMH−16−0019/2016
A/Newcastle/30/2016
A/Brazil/6359/2016
A/Argentina/943/2016
A/Bangladesh/402/2016
A/Mississippi/92hCK/2017
A/Mississippi/49hCK/2017
A/Mississippi/62hCK/2017
A/Mississippi/15/2017
A/Mississippi/89hCK/2017
A/Mississippi/8hCK/2017
A/Mississippi/13/2017
A/Mississippi/13hCK/2017
A/Mississippi/258hCK/2017
A/Mississippi/95/2017
A/Mississippi/12hCK/2017
A/Mississippi/51/2017
A/Mississippi/144hCK/2017
A/Mississippi/62/2017
A/Mississippi/25/2017
A/Mississippi/93/2017
A/Mississippi/12/2017
A/Mississippi/85hCK/2017
A/Mississippi/79hCK/2017
A/Mississippi/78/2017
A/Mississippi/78hCK/2017
A/Mississippi/79/2017
A/Mississippi/89/2017
A/Mississippi/59/2017
A/Mississippi/57hCK/2017
A/Mississippi/58/2017
A/Mississippi/58hCK/2017
A/Mississippi/42hCK/2017
A/Mississippi/48/2017
A/Mississippi/102/2017
A/Mississippi/102hCK/2017
A/Mississippi/101/2017
A/Mississippi/100/2017
A/Mississippi/101hCK/2017
A/Mississippi/2/2017
A/Michigan/399/2018
A/Cote_DIvoire/1549/2017
A/Mississippi/28hCK/2017
A/Mississippi/28/2017
A/China/61252/2014
A/Michigan/15/2014
A/Colorado/7205/2018
A/Washington/S27181/2018
A/England/538/2018
A/Baltimore/T0101/2017
A/Peru/4617/2017
A/Hong_Kong/4801/2014
A/Texas/178/2016
A/Switzerland/9715293/2013
A/Stockholm/6/2014
A/Texas/50/2012
A/Victoria/361/2011
A/Perth/16/2009
2008
2009
2010
2011
2012
2013
2014
2015
2016
2017
2018
Collection Year

FIG. S1d NP

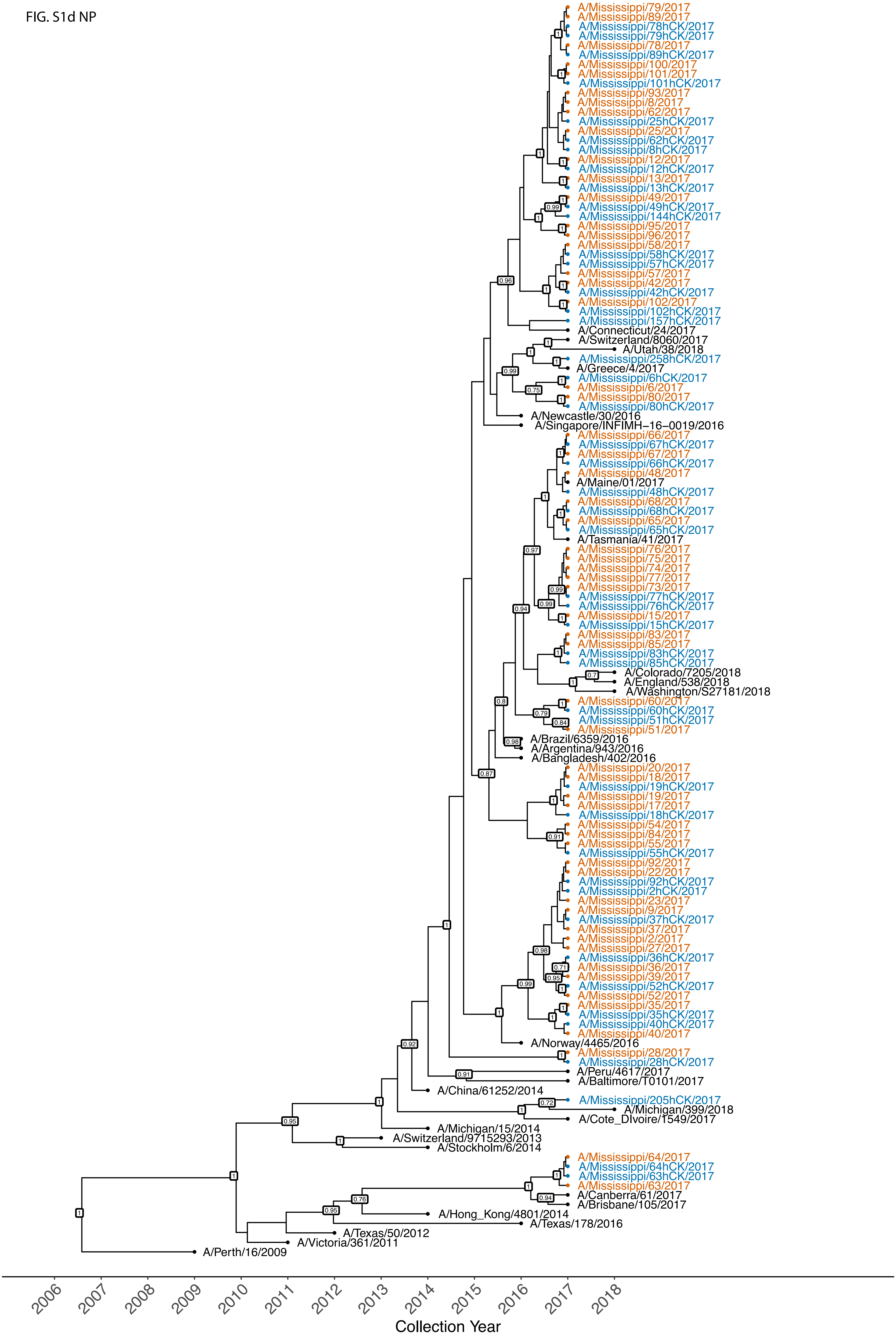

A/Mississippi/79/2017
A/Mississippi/89/2017
A/Mississippi/78hCK/2017
A/Mississippi/79hCK/2017
A/Mississippi/78/2017
A/Mississippi/89hCK/2017
A/Mississippi/100/2017
A/Mississippi/101/2017
A/Mississippi/101hCK/2017
A/Mississippi/93/2017
A/Mississippi/8/2017
A/Mississippi/62/2017
A/Mississippi/25hCK/2017
A/Mississippi/25/2017
A/Mississippi/62hCK/2017
A/Mississippi/8hCK/2017
A/Mississippi/12/2017
A/Mississippi/12hCK/2017
A/Mississippi/13/2017
A/Mississippi/13hCK/2017
A/Mississippi/49/2017
A/Mississippi/49hCK/2017
A/Mississippi/144hCK/2017
A/Mississippi/95/2017
A/Mississippi/96/2017
A/Mississippi/58/2017
A/Mississippi/58hCK/2017
A/Mississippi/57hCK/2017
A/Mississippi/57/2017
A/Mississippi/42/2017
A/Mississippi/42hCK/2017
A/Mississippi/102/2017
A/Mississippi/102hCK/2017
A/Mississippi/157hCK/2017
A/Connecticut/24/2017
A/Switzerland/8060/2017
A/Utah/38/2018
A/Mississippi/258hCK/2017
A/Greece/4/2017
A/Mississippi/6hCK/2017
A/Mississippi/6/2017
A/Mississippi/80/2017
A/Mississippi/80hCK/2017
A/Newcastle/30/2016
A/Singapore/INFIMH-16-0019/2016
A/Mississippi/66/2017
A/Mississippi/67hCK/2017
A/Mississippi/67/2017
A/Mississippi/66hCK/2017
A/Mississippi/48/2017
A/Maine/01/2017
A/Mississippi/48hCK/2017
A/Mississippi/68/2017
A/Mississippi/68hCK/2017
A/Mississippi/65/2017
A/Mississippi/65hCK/2017
A/Tasmania/41/2017
A/Mississippi/76/2017
A/Mississippi/75/2017
A/Mississippi/74/2017
A/Mississippi/77/2017
A/Mississippi/73/2017
A/Mississippi/77hCK/2017
A/Mississippi/76hCK/2017
A/Mississippi/15/2017
A/Mississippi/15hCK/2017
A/Mississippi/83/2017
A/Mississippi/85/2017
A/Mississippi/83hCK/2017
A/Mississippi/85hCK/2017
A/Colorado/7205/2018
A/England/538/2018
A/Washington/S27181/2018
A/Mississippi/60/2017
A/Mississippi/60hCK/2017
A/Mississippi/51hCK/2017
A/Mississippi/51/2017
A/Brazil/6359/2016
A/Argentina/943/2016
A/Bangladesh/402/2016
A/Mississippi/20/2017
A/Mississippi/18/2017
A/Mississippi/19hCK/2017
A/Mississippi/19/2017
A/Mississippi/17/2017
A/Mississippi/18hCK/2017
A/Mississippi/54/2017
A/Mississippi/84/2017
A/Mississippi/55/2017
A/Mississippi/55hCK/2017
A/Mississippi/92/2017
A/Mississippi/22/2017
A/Mississippi/92hCK/2017
A/Mississippi/2hCK/2017
A/Mississippi/23/2017
A/Mississippi/9/2017
A/Mississippi/37hCK/2017
A/Mississippi/37/2017
A/Mississippi/2/2017
A/Mississippi/27/2017
A/Mississippi/36hCK/2017
A/Mississippi/36/2017
A/Mississippi/39/2017
A/Mississippi/52hCK/2017
A/Mississippi/52/2017
A/Mississippi/35/2017
A/Mississippi/35hCK/2017
A/Mississippi/40hCK/2017
A/Mississippi/40/2017
A/Norway/4465/2016
A/Mississippi/28/2017
A/Mississippi/28hCK/2017
A/Peru/4617/2017
A/Baltimore/T0101/2017
A/China/61252/2014
A/Mississippi/205hCK/2017
A/Michigan/399/2018
A/Cote_DIvoire/1549/2017
A/Michigan/15/2014
A/Switzerland/9715293/2013
A/Stockholm/6/2014
A/Mississippi/64/2017
A/Mississippi/64hCK/2017
A/Mississippi/63hCK/2017
A/Mississippi/63/2017
A/Canberra/61/2017
A/Brisbane/105/2017
A/Hong_Kong/4801/2014
A/Texas/178/2016
A/Texas/50/2012
A/Victoria/361/2011
A/Perth/16/2009
2006
2007
2008
2009
2010
2011
2012
2013
2014
2015
2016
2017
2018
Collection Year

FIG. S1e MP

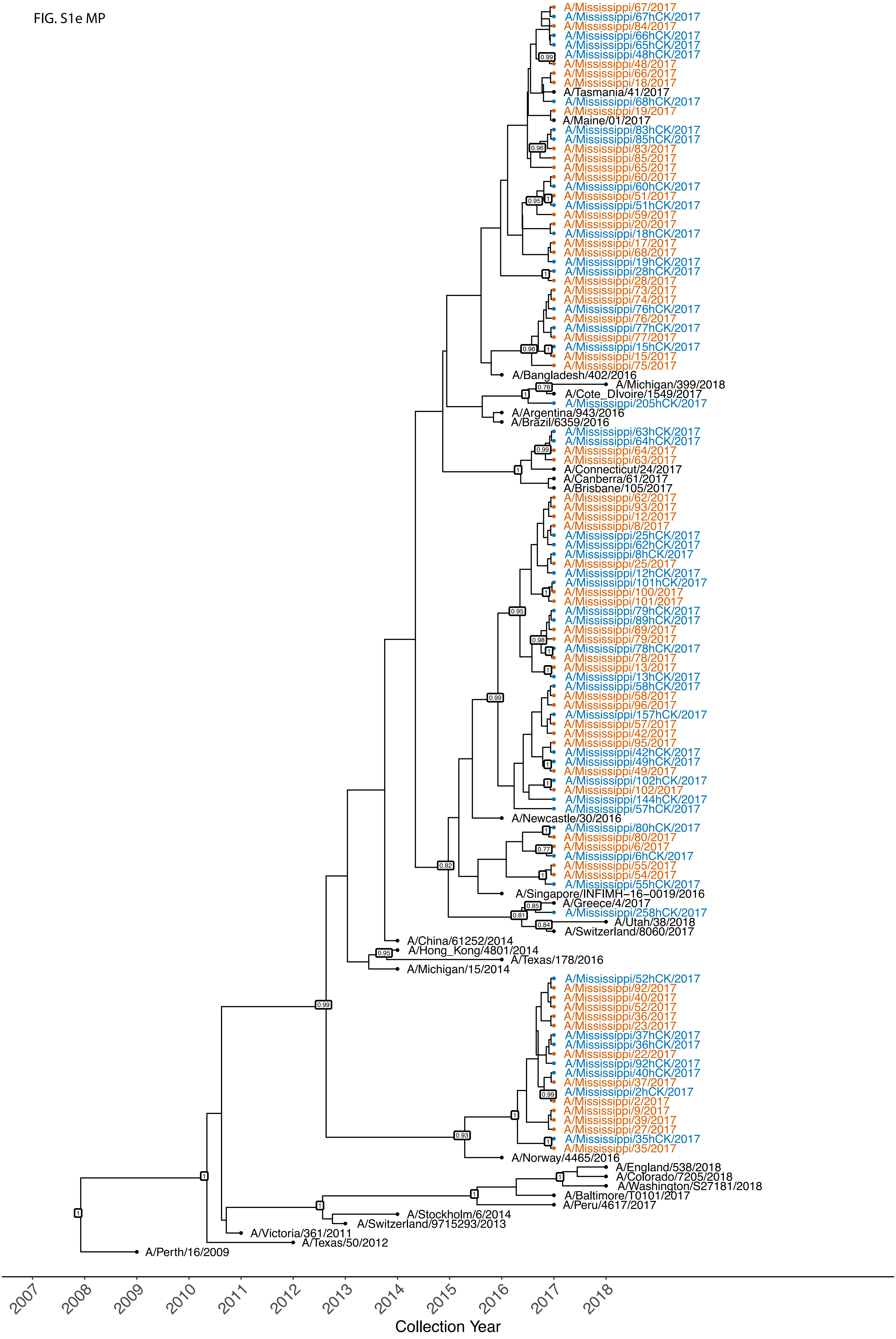

A/Mississippi/67/2017
A/Mississippi/67hCK/2017
A/Mississippi/84/2017
A/Mississippi/66hCK/2017
A/Mississippi/65hCK/2017
A/Mississippi/48hCK/2017
A/Mississippi/48/2017
A/Mississippi/66/2017
A/Mississippi/18/2017
A/Tasmania/41/2017
A/Mississippi/68hCK/2017
A/Mississippi/19/2017
A/Maine/01/2017
A/Mississippi/83hCK/2017
A/Mississippi/85hCK/2017
A/Mississippi/83/2017
A/Mississippi/85/2017
A/Mississippi/65/2017
A/Mississippi/60/2017
A/Mississippi/60hCK/2017
A/Mississippi/51/2017
A/Mississippi/51hCK/2017
A/Mississippi/59/2017
A/Mississippi/20/2017
A/Mississippi/18hCK/2017
A/Mississippi/17/2017
A/Mississippi/68/2017
A/Mississippi/19hCK/2017
A/Mississippi/28hCK/2017
A/Mississippi/28/2017
A/Mississippi/73/2017
A/Mississippi/74/2017
A/Mississippi/76hCK/2017
A/Mississippi/76/2017
A/Mississippi/77hCK/2017
A/Mississippi/77/2017
A/Mississippi/15hCK/2017
A/Mississippi/15/2017
A/Mississippi/75/2017
A/Bangladesh/402/2016
A/Michigan/399/2018
A/Cote_DIvoire/1549/2017
A/Mississippi/205hCK/2017
A/Argentina/943/2016
A/Brazil/6359/2016
A/Mississippi/63hCK/2017
A/Mississippi/64hCK/2017
A/Mississippi/64/2017
A/Mississippi/63/2017
A/Connecticut/24/2017
A/Canberra/61/2017
A/Brisbane/105/2017
A/Mississippi/62/2017
A/Mississippi/93/2017
A/Mississippi/12/2017
A/Mississippi/8/2017
A/Mississippi/25hCK/2017
A/Mississippi/62hCK/2017
A/Mississippi/8hCK/2017
A/Mississippi/25/2017
A/Mississippi/12hCK/2017
A/Mississippi/101hCK/2017
A/Mississippi/100/2017
A/Mississippi/101/2017
A/Mississippi/79hCK/2017
A/Mississippi/89hCK/2017
A/Mississippi/89/2017
A/Mississippi/79/2017
A/Mississippi/78hCK/2017
A/Mississippi/78/2017
A/Mississippi/13/2017
A/Mississippi/13hCK/2017
A/Mississippi/58hCK/2017
A/Mississippi/58/2017
A/Mississippi/96/2017
A/Mississippi/157hCK/2017
A/Mississippi/57/2017
A/Mississippi/42/2017
A/Mississippi/95/2017
A/Mississippi/42hCK/2017
A/Mississippi/49hCK/2017
A/Mississippi/49/2017
A/Mississippi/102hCK/2017
A/Mississippi/102/2017
A/Mississippi/144hCK/2017
A/Mississippi/57hCK/2017
A/Newcastle/30/2016
A/Mississippi/80hCK/2017
A/Mississippi/80/2017
A/Mississippi/6/2017
A/Mississippi/6hCK/2017
A/Mississippi/55/2017
A/Mississippi/54/2017
A/Mississippi/55hCK/2017
A/Singapore/INFIMH-16-0019/2016
A/Greece/4/2017
A/Mississippi/258hCK/2017
A/Utah/38/2018
A/Switzerland/8060/2017
A/China/61252/2014
A/Hong_Kong/4801/2014
A/Texas/178/2016
A/Michigan/15/2014
A/Mississippi/52hCK/2017
A/Mississippi/92/2017
A/Mississippi/40/2017
A/Mississippi/52/2017
A/Mississippi/36/2017
A/Mississippi/23/2017
A/Mississippi/37hCK/2017
A/Mississippi/36hCK/2017
A/Mississippi/22/2017
A/Mississippi/92hCK/2017
A/Mississippi/40hCK/2017
A/Mississippi/37/2017
A/Mississippi/2hCK/2017
A/Mississippi/2/2017
A/Mississippi/9/2017
A/Mississippi/39/2017
A/Mississippi/27/2017
A/Mississippi/35hCK/2017
A/Mississippi/35/2017
A/Norway/4465/2016
A/England/538/2018
A/Colorado/7205/2018
A/Washington/S27181/2018
A/Baltimore/T0101/2017
A/Peru/4617/2017
A/Stockholm/6/2014
A/Switzerland/9715293/2013
A/Victoria/361/2011
A/Texas/50/2012
A/Perth/16/2009
0.99
0.96
0.95
1
0.96
0.76
0.99
0.95
0.98
0.99
0.77
0.82
0.85
0.81
0.84
0.95
0.99
0.93
2007
2008
2009
2010
2011
2012
2013
2014
2015
2016
2017
2018
Collection Year

FIG. S1f NS

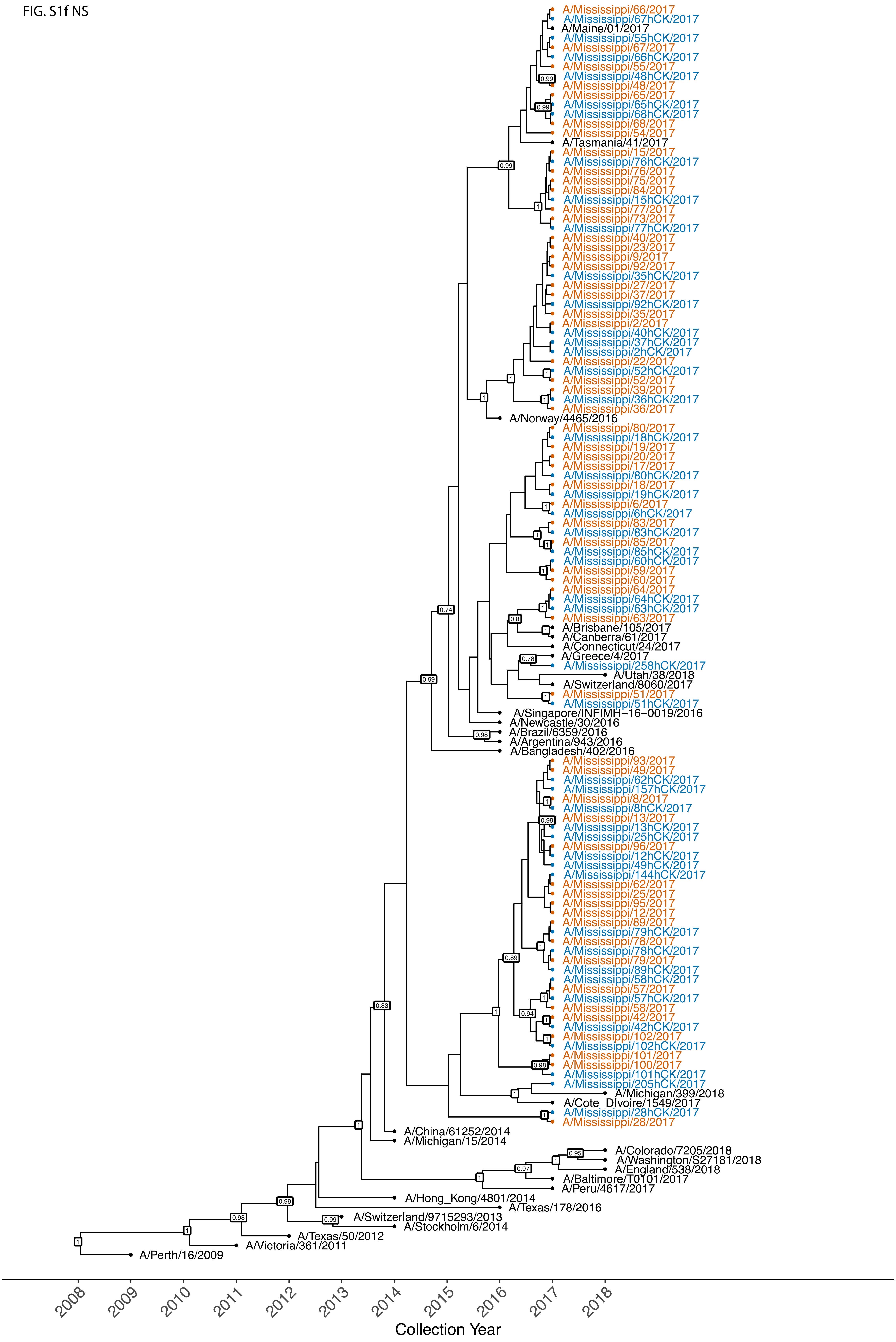
A/Mississippi/66/2017
A/Mississippi/67hCK/2017
A/Maine/01/2017
A/Mississippi/55hCK/2017
A/Mississippi/67/2017
A/Mississippi/66hCK/2017
A/Mississippi/55/2017
A/Mississippi/48hCK/2017
A/Mississippi/48/2017
A/Mississippi/65/2017
A/Mississippi/65hCK/2017
A/Mississippi/68hCK/2017
A/Mississippi/68/2017
A/Mississippi/54/2017
A/Tasmania/41/2017
A/Mississippi/15/2017
A/Mississippi/76hCK/2017
A/Mississippi/76/2017
A/Mississippi/75/2017
A/Mississippi/84/2017
A/Mississippi/15hCK/2017
A/Mississippi/77/2017
A/Mississippi/73/2017
A/Mississippi/77hCK/2017
A/Mississippi/40/2017
A/Mississippi/23/2017
A/Mississippi/9/2017
A/Mississippi/92/2017
A/Mississippi/35hCK/2017
A/Mississippi/27/2017
A/Mississippi/37/2017
A/Mississippi/92hCK/2017
A/Mississippi/35/2017
A/Mississippi/2/2017
A/Mississippi/40hCK/2017
A/Mississippi/37hCK/2017
A/Mississippi/2hCK/2017
A/Mississippi/22/2017
A/Mississippi/52hCK/2017
A/Mississippi/52/2017
A/Mississippi/39/2017
A/Mississippi/36hCK/2017
A/Mississippi/36/2017
A/Norway/4465/2016
A/Mississippi/80/2017
A/Mississippi/18hCK/2017
A/Mississippi/19/2017
A/Mississippi/20/2017
A/Mississippi/17/2017
A/Mississippi/80hCK/2017
A/Mississippi/18/2017
A/Mississippi/19hCK/2017
A/Mississippi/6/2017
A/Mississippi/6hCK/2017
A/Mississippi/83/2017
A/Mississippi/83hCK/2017
A/Mississippi/85/2017
A/Mississippi/85hCK/2017
A/Mississippi/60hCK/2017
A/Mississippi/59/2017
A/Mississippi/60/2017
A/Mississippi/64/2017
A/Mississippi/64hCK/2017
A/Mississippi/63hCK/2017
A/Mississippi/63/2017
A/Brisbane/105/2017
A/Canberra/61/2017
A/Connecticut/24/2017
A/Greece/4/2017
A/Mississippi/258hCK/2017
A/Utah/38/2018
A/Switzerland/8060/2017
A/Mississippi/51/2017
A/Mississippi/51hCK/2017
A/Singapore/INFIMH-16-0019/2016
A/Newcastle/30/2016
A/Brazil/6359/2016
A/Argentina/943/2016
A/Bangladesh/402/2016
A/Mississippi/93/2017
A/Mississippi/49/2017
A/Mississippi/62hCK/2017
A/Mississippi/157hCK/2017
A/Mississippi/8/2017
A/Mississippi/8hCK/2017
A/Mississippi/13/2017
A/Mississippi/13hCK/2017
A/Mississippi/25hCK/2017
A/Mississippi/96/2017
A/Mississippi/12hCK/2017
A/Mississippi/49hCK/2017
A/Mississippi/144hCK/2017
A/Mississippi/62/2017
A/Mississippi/25/2017
A/Mississippi/95/2017
A/Mississippi/12/2017
A/Mississippi/89/2017
A/Mississippi/79hCK/2017
A/Mississippi/78/2017
A/Mississippi/78hCK/2017
A/Mississippi/79/2017
A/Mississippi/89hCK/2017
A/Mississippi/58hCK/2017
A/Mississippi/57/2017
A/Mississippi/57hCK/2017
A/Mississippi/58/2017
A/Mississippi/42/2017
A/Mississippi/42hCK/2017
A/Mississippi/102/2017
A/Mississippi/102hCK/2017
A/Mississippi/101/2017
A/Mississippi/100/2017
A/Mississippi/101hCK/2017
A/Mississippi/205hCK/2017
A/Michigan/399/2018
A/Cote_DIvoire/1549/2017
A/Mississippi/28hCK/2017
A/Mississippi/28/2017
A/China/61252/2014
A/Michigan/15/2014
A/Colorado/7205/2018
A/Washington/S27181/2018
A/England/538/2018
A/Baltimore/T0101/2017
A/Peru/4617/2017
A/Hong_Kong/4801/2014
A/Texas/178/2016
A/Switzerland/9715293/2013
A/Stockholm/6/2014
A/Texas/50/2012
A/Victoria/361/2011
A/Perth/16/2009
2008
2009
2010
2011
2012
2013
2014
2015
2016
2017
2018
Collection Year

Supplementary Figure 2

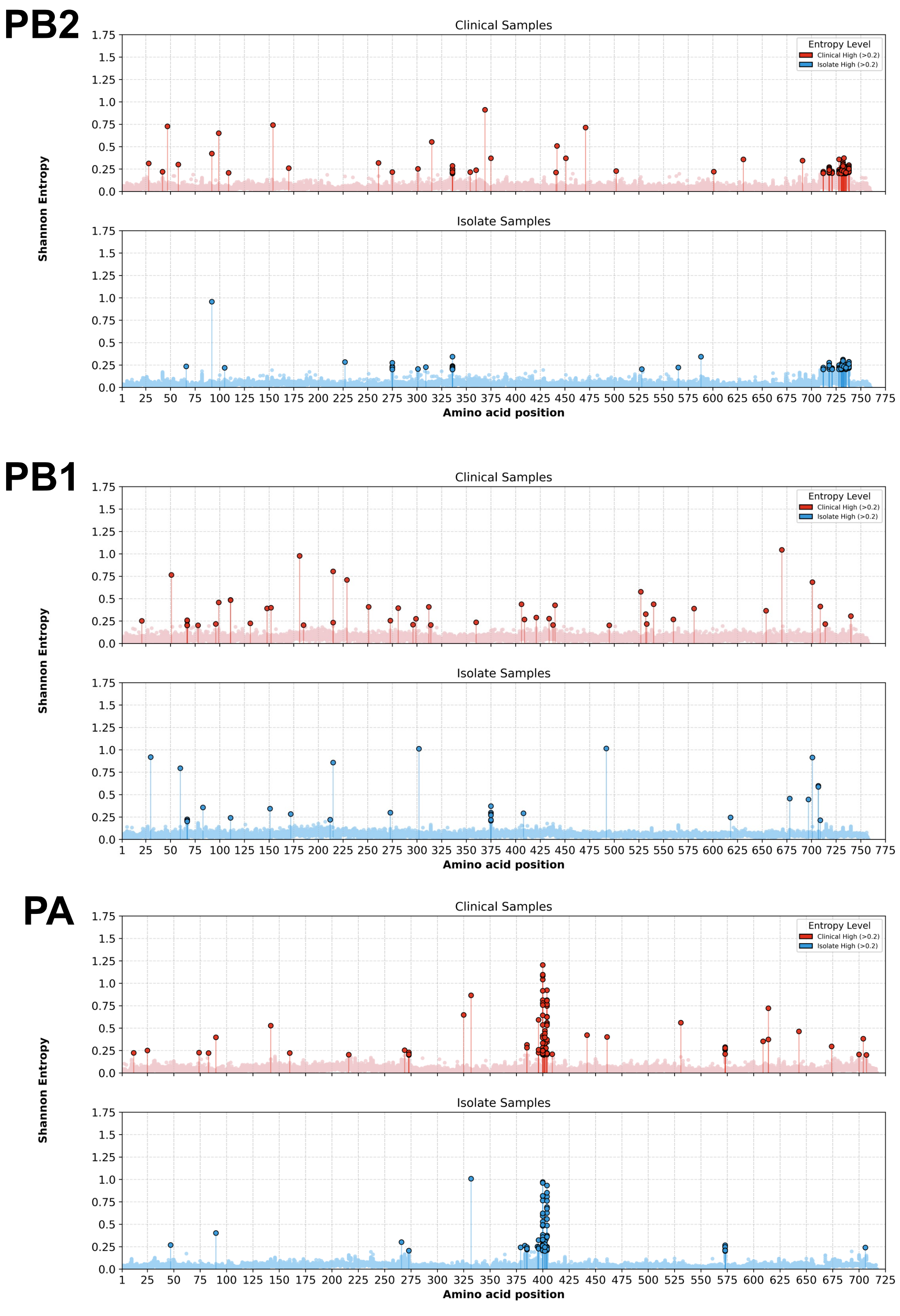

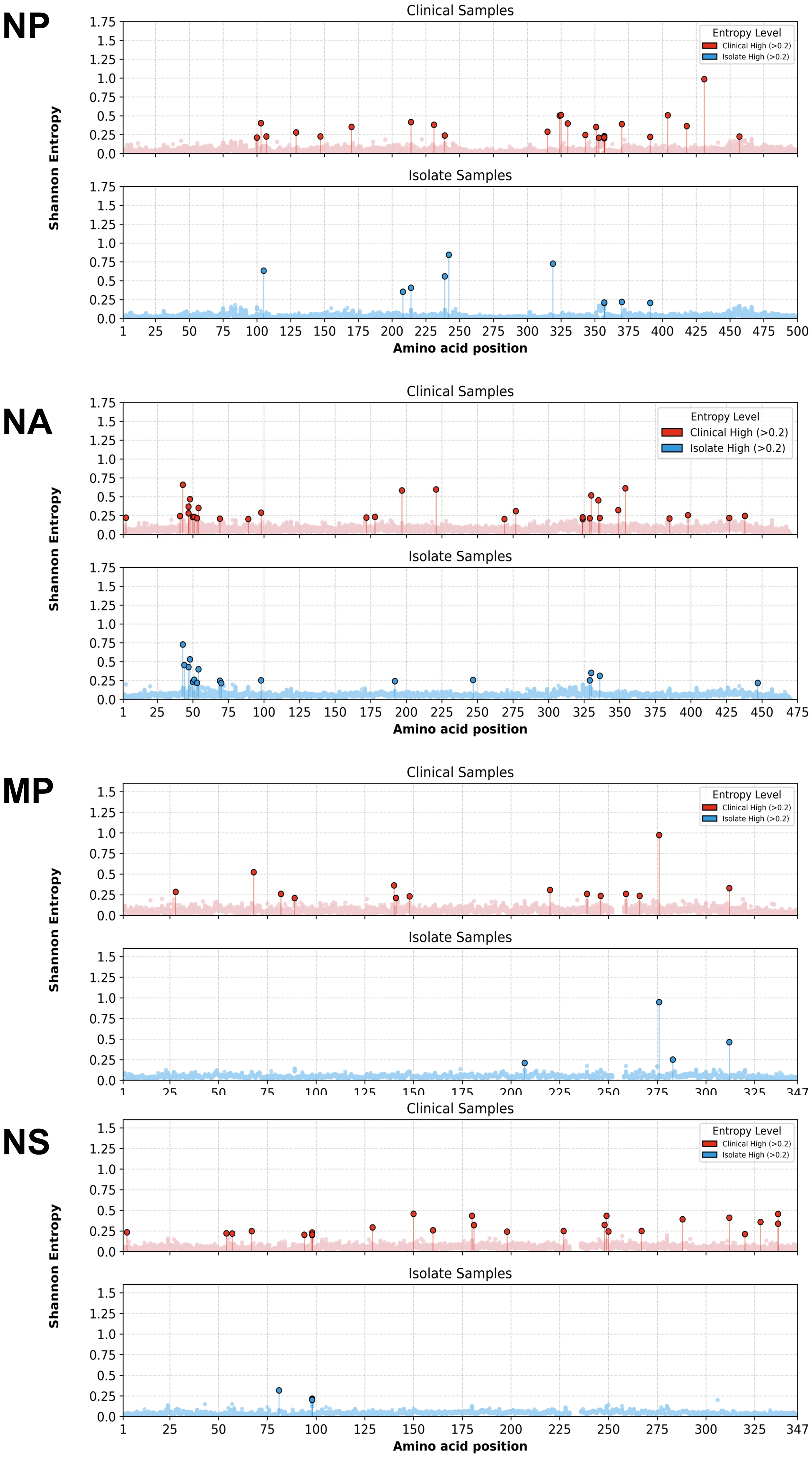
NP
Clinical Samples
Isolate Samples
Shannon Entropy
Amino acid position
Entropy Level
Clinical High (>0.2)
Isolate High (>0.2)
1.75
1.5
1.25
1.0
0.75
0.5
0.25
0.0
1
25
50
75
100
125
150
175
200
225
250
275
300
325
350
375
400
425
450
475
500
NA
Clinical Samples
Isolate Samples
Shannon Entropy
Amino acid position
Entropy Level
Clinical High (>0.2)
Isolate High (>0.2)
1
25
50
75
100
125
150
175
200
225
250
275
300
325
350
375
400
425
450
475
MP
Clinical Samples
Isolate Samples
Shannon Entropy
Entropy Level
Clinical High (>0.2)
Isolate High (>0.2)
NS
Clinical Samples
Isolate Samples
Shannon Entropy
Amino acid position
Entropy Level
Clinical High (>0.2)
Isolate High (>0.2)
1
25
50
75
100
125
150
175
200
225
250
275
300
325
347
